# Integration Technology of Micro-LED for Next-Generation Display

**DOI:** 10.34133/research.0047

**Published:** 2023-04-12

**Authors:** Dingbo Chen, Yu-Chang Chen, Guang Zeng, David Wei Zhang, Hong-Liang Lu

**Affiliations:** ^1^ State Key Laboratory of ASIC and System, Shanghai Institute of Intelligent Electronics & Systems, School of Microelectronics, Fudan University, Shanghai 200433, China.; ^2^ Jia Shan Fudan Institute, Jiaxing, Zhejiang Province 314100, China.; ^3^ Key Laboratory of Specialty Fiber Optics and Optical Access Networks, Shanghai Institute Communication and Data Science, Shanghai University, Shanghai 200444, China.

## Abstract

Inorganic micro light-emitting diodes (micro-LEDs) based on III-V compound semiconductors have been widely studied for self-emissive displays. From chips to applications, integration technology plays an indispensable role in micro-LED displays. For example, large-scale display relies on the integration of discrete device dies to achieve extended micro-LED array, and full color display requires integration of red, green, and blue micro-LED units on the same substrate. Moreover, the integration with transistors or complementary metal-oxide-semiconductor circuits are necessary to control and drive the micro-LED display system. In this review article, we summarized the 3 main integration technologies for micro-LED displays, which are called transfer integration, bonding integration, and growth integration. An overview of the characteristics of these 3 integration technologies is presented, while various strategies and challenges of integrated micro-LED display system are discussed.

## Introduction

The concept of micro light-emitting diode (micro-LED) was put forward in 2000 [1,2], and extensive scientific research on micro-LED has been revitalizing the LED industry for more than 2 decades [3–22]. Benefiting from the advantages of traditional LED, micro-LED display based on III-V compound semiconductors possesses higher efficiency, lower energy consumption than liquid crystal display, higher brightness, and longer lifetime than organic LED display [23–28]. Therefore, micro-LED display has been considered as a promising candidate for the next-generation display technology [26,29,30]. Unlike conventional large-area LEDs for lighting, micro-LEDs have typical pixel (micro-LED die) sizes below 100 μm or even 50 μm. Such a small size brings great challenges to the array arrangement and system integration of micro-LED displays. At present, integration technology has become the most critical problem in the development of high-performance micro-LED displays.

Specifically, the role of integration technology in micro-LED display manufacturing is reflected in the following 3 aspects. Firstly, full-color display requires integrated micro-LED array with polychromatic emission (red, green, blue [RGB]), or integration of monochromatic micro-LED with color convertors [31,32]. Based on different full-color schemes, the integration method is either transferring RGB onto an assembled package or growing RGB together into a same chip. Secondly, it was necessary to integrate pixel array with a driving circuit for addressing pixels passively or actively [8,10]. The driving circuit module is integrated with micro-LED through pick-and-place, bonding, or metal interconnection to form a two-dimensional (2D) or three-dimensional integrated structure [21,33]. Thirdly, in order to obtain enhanced performance and expanded functionalities, intelligent display is pursued through heterogeneous integration with other functional devices and components [34,35]. Similar to the scale-down approach of silicon complementary metal-oxide-semiconductor (CMOS) circuits or thin-film transistor (TFT) circuits, numerous integration techniques have been developed to fabricate full-color, high-resolution, and multifunctional micro-LED display systems with more compact dimensions.

**Fig. 1.**
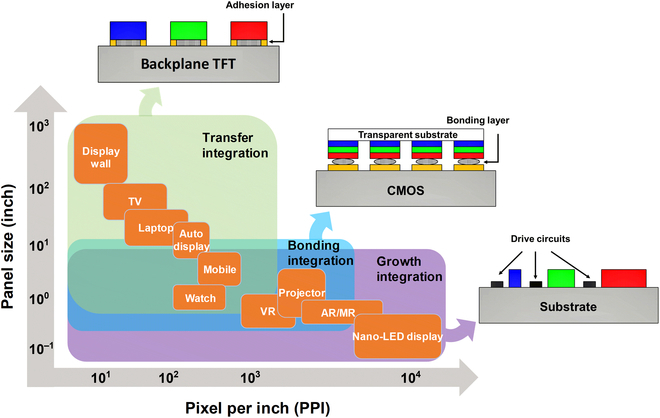
The main application scenarios of micro-LED display and their characteristic display area and pixel density. The insets are corresponding schematic diagrams of micro-LED subpixels fabricated by transfer integration, bonding integration, and growth integration.

From the perspective of manufacturing process, the integration of micro-LED displays can be summarized into 3 categories: transfer integration, bonding integration, and growth integration. The transfer integration is integrating several discrete devices in a package with wire bonding or metal connects. In the micro-LED display technology, the transfer integration is used to assemble the micro-LED dies on the receiver substrate, followed by forming electrical interconnects [36]. The bonding integration is a common heterogeneous integration in traditional semiconductor devices. Wafer bonding can be used for integrated devices or materials in the micro-LED display system [37]. The so-called monolithic hybrid, which is achieved through a flip-chip process, can realize high-resolution matrix-addressable micro-LED display [22]. The growth integration means that the materials making up the system are all grown on the same substrate. The integrated modules for micro-LED display go through a full monolithic process. Growth integration works for both homogeneous devices and heterogeneous devices in horizontal or vertical forms. Selective epi removal (SER) and selective area growth (SAG) are regarded as 2 feasible schemes for the growth integration of micro-LED displays [38]. Different process conditions of above 3 integration technologies qualified their own structural characteristics and application tendencies. Figure 1 summarizes a series of micro-LED application scenarios, as well as their characteristic display area (panel size) and pixel density (pixel per inch [PPI]). The insets show schematic diagrams of micro-LED pixels prepared by 3 types of integrations mentioned above.

Over the past few years, many review papers about micro-LED have gradually emerged, which cover material preparation, device optimization, and application development of micro-LED [18,19,26–32,39–41]. However, unrelated to the science of materials and devices, detailed review upon the integration technology was unique to the field of the science of micro-LED displays and yet lacking. In the following sections, we present an overview of transfer integration, bonding integration, and growth integration of micro-LED in turn. Finally, the micro-LED integration and its future development are summarized and prospected.

## Transfer Integration of Micro-LED

Manufacturing a large-scale high-definition micro-LED display screen, such as 55″ 4K TV, requires assembling millions of LED dies. Therefore, transfer integration of micro-LED is usually called a mass transfer, i.e., the so-called pick-and-place process. Pioneer companies and research institutes around the world have provided various paths for this process based on different physical mechanisms. For instance, as summarized in previous reviews, the method of transfer printing was developed utilizing van der Waals force between LEDs and elastomer stamp or roll stamp [42–44]. Laser selective-release transfer was performed owning to the gravity and expansion force [45,46]. The electrostatic [47,48] and electromagnetic [49] pick-up transfer was by means of electrostatic force and magnetic force generated on the transfer head or transfer arm, respectively. In addition, fluidic assembly via gravity and capillary forces was also been investigated for mass transfer [50,51]. What this section presents is not enumeration and description of each pick-and-place method as in previous reviews, but overall introduction of transfer integration. As a form of heterogeneous integration, transfer integration consists of 3 technical steps: substrate release, pick-and-place, and electrical interconnection.

### Substrate release of micro-LED array

**Fig. 2.**
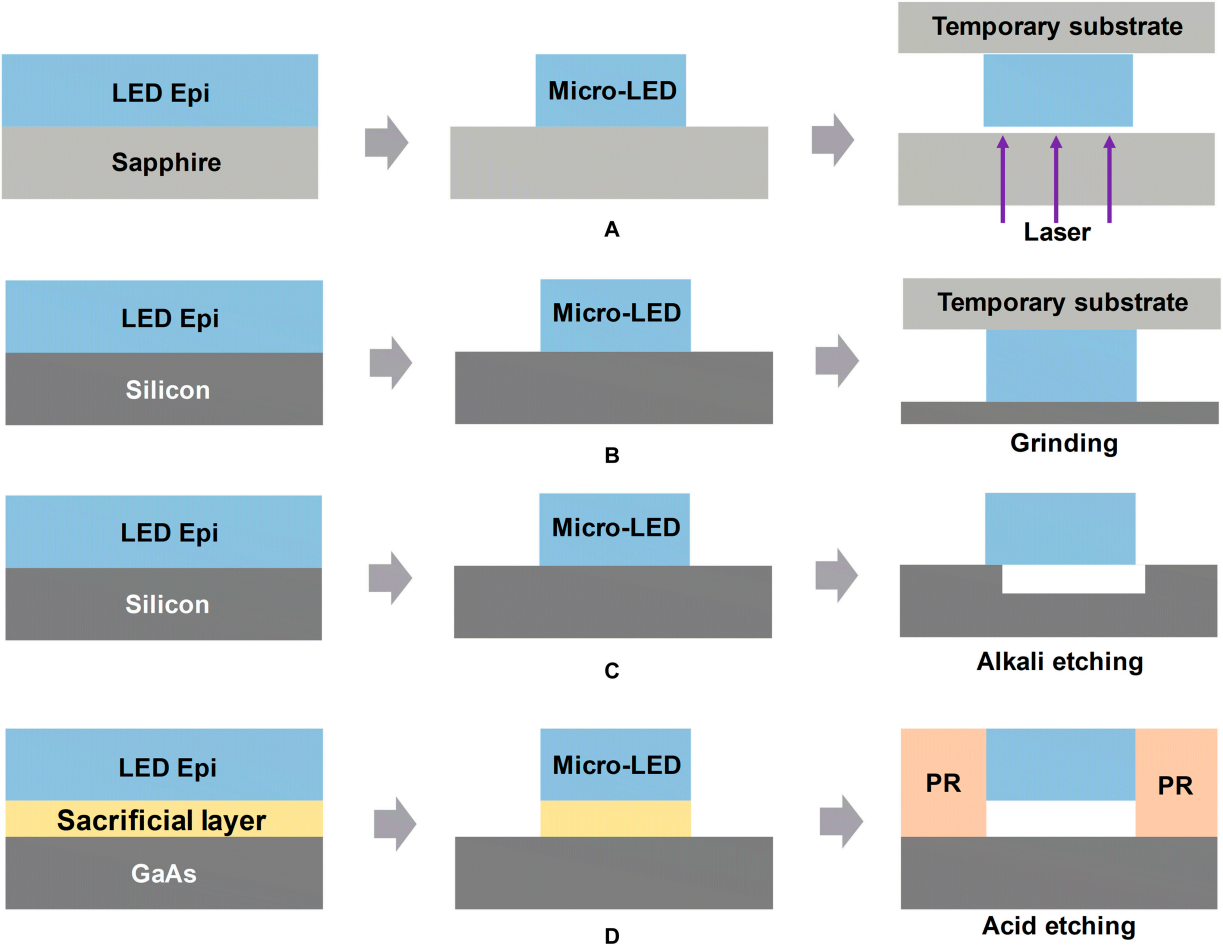
Schematic illustration of the processes of substrate release by (A) LLO, (B) mechanical grinding, and wet chemical etching using (C) alkali and (D) acid solutions.

Commonly, GaN-based micro-LEDs are grown and fabricated on silicon or sapphire substrate and AlGaInP-based micro-LED on GaAs substrate. The grown substrates often have a thickness of hundreds of microns. Such a large thickness will bring trouble to the electrical interconnection and thermal management of the transferred micro-LEDs. Therefore, substrate release or removal is a prerequisite for almost all picking and transferring processes. Besides, the choice of substrate release techniques needs to take physical and chemical properties of corresponding material into consideration. Figure 2 shows common substrate release techniques used by different donor substrates. Generally speaking, the growth substrate of micro-LED can be released physically and chemically. The physical methods include laser lift-off (LLO) and mechanical grinding techniques. The chemical methods include acid or alkali wet etching.

In 1996, Kelly et al. [52] found that short laser pulse can induce the decomposition of GaN with high spatial resolution, and they used a 355-nm pulsed laser to realize the lift-off of GaN film on sapphire substrate. When the laser passes through sapphire, its energy is absorbed by a GaN epitaxial layer, and then a thin layer of GaN decomposes into liquid Ga and N_2_ gas. Finally, the stress generated by the decomposition products at the interface promotes the release of substrate. According to the above theory, in 1999, Wong et al. [53,54] fabricated the thin-film InGaN LED membranes by LLO. Since then, LLO technique has been widely used for substrate removal of LEDs and micro-LEDs [55]. It should be noted that, in order to achieve conformal array transfer, the micro-LED wafer needs to be adhered to a temporary wafer or transfer tape in advance.

However, LLO technique only works with an ultraviolet (UV)-transparent substrate such as sapphire substrate. For Si or GaAs substrate, mechanical grinding and wet chemical etching techniques are viable options. During the mechanical grinding process, the micro-LED will be severely mechanically impacted, so it needs to form a firm contact with the temporary substrate, which will bring trouble to the subsequent transfer. Therefore, the mechanical grinding release process is mainly used to realize the substrate transfer of vertical structure LEDs. Dawson et al. [56] used KOH solution to anisotropically etch Si to achieve substrate release, as shown in Fig. 2C. By defining the supporting anchors during inductively coupled plasma mesa etching, the micro-LED platelets were tied and suspended in its original position after undercut etching, and transferable micro-LED array was already formed.

Since GaAs substrates do not have anisotropic corrosion characteristics, the substrate release process of AlGaInP-based red-emitting micro-LED is different from that of GaN-based devices. Rogers et al. [57,58] introduced an epitaxial insertion layer of AlAs on the GaAs wafer that serves as active or sacrificial materials. Through selectively etching the sacrificial layer using hydrofluoric acid, the micro-LED array was completely released from growth substrate. In this case, the photoresist is used to anchor the micro-LED die in the fixed position until the transfer step. It was noted that the anchors made of silicon or photoresist are vulnerable, and the micro-LED can be picked up easily.

### Picking and placing of micro-LED

After substrate release, picking and placing were conducted. In this process, high transfer speed and high transfer precision are 2 key technical parameters to realize low-cost and high-resolution display. Here, we mainly discussed elastomer stamp and laser selective release, which were proven to have outstanding transfer performance [31,36].

**Fig. 3.**
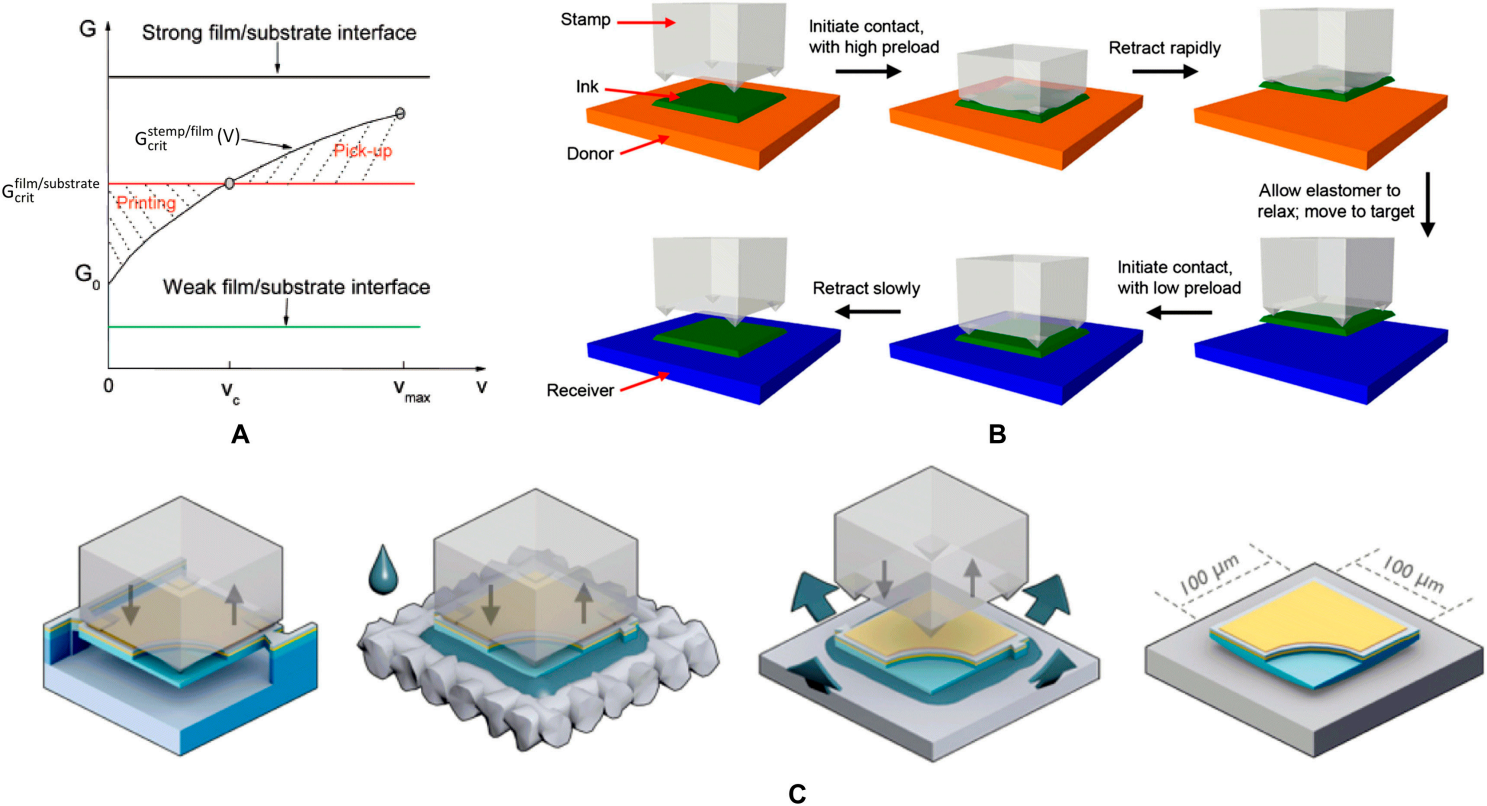
(A) Schematic illustrating stamp velocity-dependent energy-release rate. There is a critical velocity between printing and pick-up [59]. (B) Schematic diagram of transfer printing by microstructured polydimethylsiloxane (PDMS) stamp [61]. (C) Schematic of transfer printing using capillary bonding. The suspended device is picked up using an elastomeric stamp, and the bottom of LED is compressed against an acetone-wetted cloth, printing the wetted LED on a receiver substrate. Finally, LED is bonded to a new substrate after thermal curing [62].

Elastomer stamp technique originated from Rogers group’s research on the reversible control of adhesion strength between stamp and film [59,60]. As seen in Fig. 3A, when the materials of stamp and film are determined, the energy-release rate of their interface (G_stamp/film_) is positively correlated with the peeling speed (V). Due to the peeling speed between film and substrate is around zero during transferring, the energy-release rate of the film/substrate interface (G_film/substrate_) is fixed. If G_stamp/film_ can be equal to the G_film/substrate_ at a critical peeling speed (V_crit_), the pick-up and printing step can be conducted at the speed of higher and lower than V_crit_, respectively. Based on above theory, elastomer stamp was used to transfer printing micro-LED array by kinetically controlling the adhesion strength between stamp and LED membranes. In 2009, Park et al. [58] transferred the AlInGaP-based micro-LED array onto a polyurethane substrate and a glass substrate by a flat polydimethylsiloxane (PDMS) stamp, and a deformable and semitransparent display was realized. In 2010, Kim et al. [61] fabricated a microstructured PDMS stamp that has a sharper reversibility window. As shown in Fig. 3B, 4 microtips distributed in the corner of the stamp can improve the repeatability and yield of transfer printing. Generally, an adhesion-enhancement layer was coated on receiving substrates to facilitate the printing of the micro-LED array. However, the adhesion-enhancement layers usually have poor thermal conductivities due to their organic nature, thus impeding heat dissipation of micro-LED. Meanwhile, the adhesion-enhancement layers can change the refractive index and deteriorate the luminous efficiency. To solve these problems, Trindade et al. [62] utilized a transient layer of a volatile liquid (acetone) to aid release of LEDs from transporting stamps. After the liquid is evaporated, the LEDs adhered to the receiving substrate by capillary bonding. The process flow was shown in Fig. 3C.

**Fig. 4.**
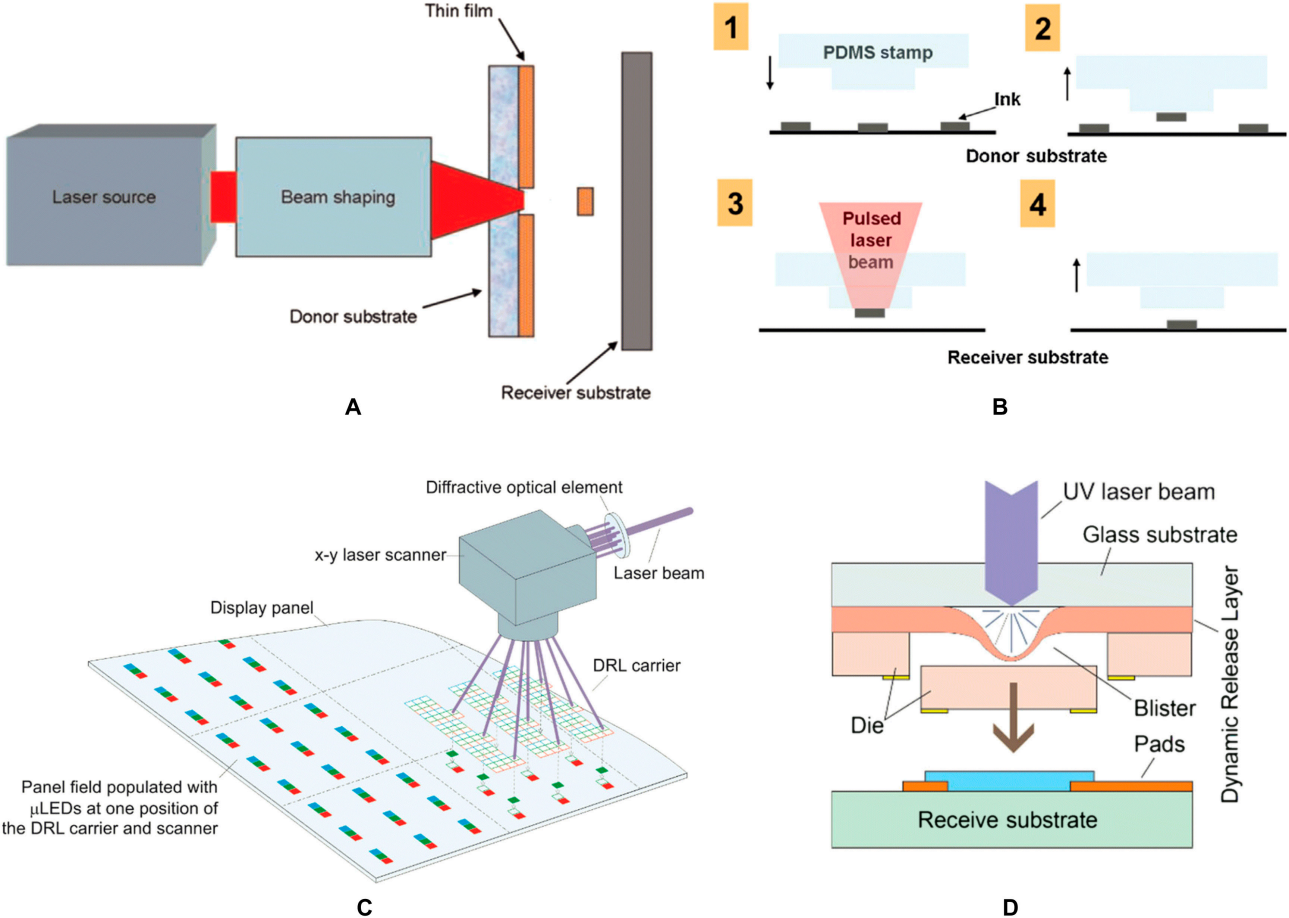
(A) Schematic description of the laser-induced forward transfer process in solid-phase film [63]. (B) Laser-assisted PDMS stamp transfer printing [67]. (C) Schematic illustration of the MPLET concept and (D) laser transfer [68].

Laser selective release was inspired by laser-induced forward transfer, which was demonstrated for the first time by Bohandy et al. [63, 64], as shown in Fig. 4A. Then, Holmes et al. [65, 66] introduced a polymer sacrificial layer, called dynamic release layer (DRL), between the substrate and the film to be transferred for the batch assembly of hybrid microelectromechanical systems. When the laser beam selectively irradiated the backside of transparent donor substrate, the DRL absorbed the energy of laser and was partially ablated. Hence, a strong local expansion or repulsive force was generated, which induced delamination of microstructure membrane and donor. Finally, the dies were transferred onto a receive substrate that was placed close to donor substrate. Saeidpourazar et al. [67] directly adopted PDMS stamp as DRL, as shown in Fig. 4B. As PDMS stamps were transparent in the infrared range, the infrared laser with wavelength 805 nm was chosen to drive the transferring of micro-LEDs from stamps to flexible substrate. When the LED was heated, the stamp–LED interface temperature rose. Due to large thermal expansion coefficient (CTE) mismatch between PDMS and LED material (α_s_ = 310 ppm/°C for PDMS and α_s_ = 2.6 ppm/°C for silicon), the thermal strain at the stamp–LED interface prompts the release of micro-LEDs. Marinov et al. [68] demonstrated a massively parallel laser-enabled transfer (MPLET) technology, in which the arrayed UV laser was used. Figure 4C shows schematic illustration of the MPLET concept. While the DRL was partially ablated, a blister was caused in DRL, as shown in Fig. 4D. With the force exerted by expanding blister and the gravity, micro-LED dies were transferred to a receive substrate across a 10- to 300-μm gap.

In general, these 2 techniques adopt the similar methods when picking the micro-LED, that is, the micro-LED is temporarily transferred to the surface of the organic thin film (such as SU-8 or benzocyclobutene) with an enhanced adhesion. The difference only exists in placing or printing. The former was placed by different van der Waals forces generated by peeling, while the latter depends on thermal expansion caused by the laser. Although this method allows transferring a large size at a high rate, the disadvantages of elastomer stamp technique are obvious. The microstructured stamps require careful engineering process and mass material consumptions. The bow of wafer and CTE mismatch between stamp and micro-LEDs can cause pitch inconsistency and even reduce yield. To some extent, laser selective release, as a noncontact transfer technique [45], made up for the shortcomings of elastomer stamp technique. It is worth mentioning that laser selective release provided benefits to fill the missing LED alone and repair the bad dies. As a result, 2 transfer techniques were compared in Table 1.

**Table 1.** Comparison of laser transfer stamp transfer to achieve transfer integration.

**Transfer method**	**Pick**	**Place**	**Advantages**	**Disadvantages**
Laser selective release	Transfer tape and stamp	Laser beam	Noncontact; high accuracy; high repeatability; easy to repair	Complex optical and equipment design
Elastomer stamp	Transfer stamp	Peeling	Large size; high speed; compatible with other electronic devices	High cost; low yield

### Interconnection of micro-LED

**Fig. 5.**
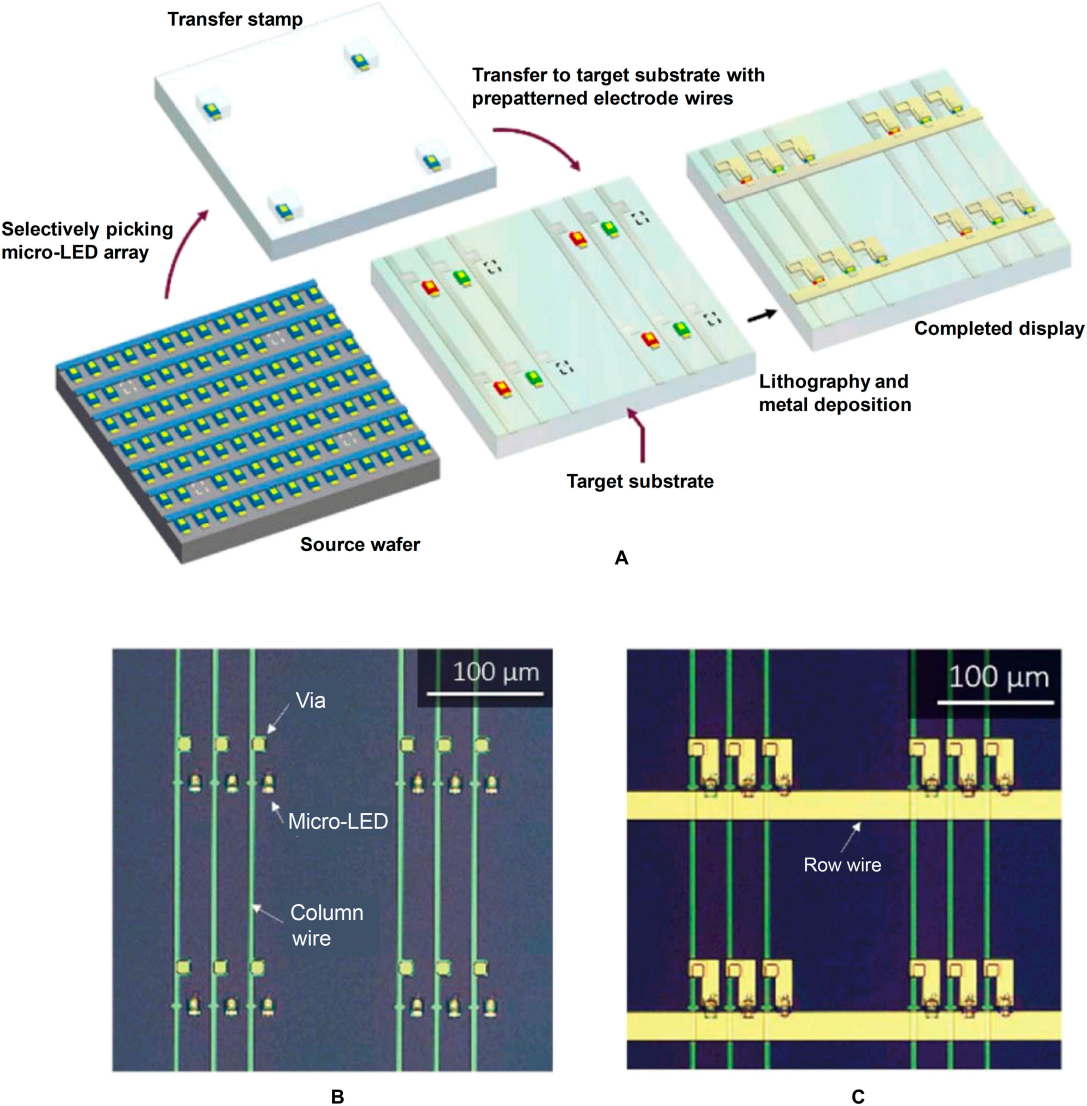
(A) Schematic illustrating interconnections of passive-matrix micro-LED display. (B) Optical micrograph taken after printing and via formation and (C) optical micrograph of the completed passive-matrix display [69].

Following the assembly process, interconnection of micro-LED dies will be conducted to realize addressable driving of micro-LED displays. After transfer printing, a metal mesh can be formed by 2 photolithographical patterns and metal deposition [57,58]. For matrix-addressable driving, the p-electrodes of each micro-LED are connected in row (or in column) and the n-electrodes of each micro-LED was connected in column (or in row). The row and column wires can be insulated with a spin-on or deposited dielectric material. Figure 5A shows a typical process of transfer integration proposed by X-Celeprint Inc. [69]. Before the red, green, and blue micro-LED dies were transferred onto the receive substrate, the column wires are previously fabricated and covered by a dielectric film. In this case, the column wires can be used as fiducial masks for the printing of micro-LED. Standard photolithography and reactive ion etching (RIE) can be used to open vias through the dielectric layers for the connection of micro-LED and column wires, as shown in Fig. 5B. Then, the electrode mesh can be achieved with only 1 metal patterning and deposition, as shown in Fig. 5C.

**Fig. 6.**
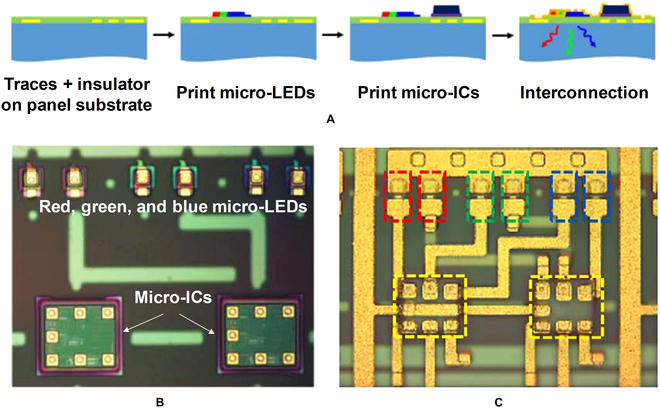
(A) Process sequence for making the active-matrix micro-LED display. (B) Optical micrograph of a single pixel after printing and (C) interconnecting [69].

However, the abovementioned interconnection mode can only obtain the passive matrix of micro-LED, the display array is generally formed on an independent insulating panel such as glass and flexible organic substrates, and the CMOS driver circuit can only be integrated with the display panel through subsequent packaging technology [58,70]. To realize active-matrix driving of micro-LED displays, micro-LEDs can be directly transferred onto substrates with micro-CMOS circuit arrays. Alternatively, the micro integrated circuit (micro-IC) unit for driving the micro-LED can also be integrated with the micro-LED by transfer printing [71,72]. Figure 6A shows the multistep transfer printing process developed by X-Celeprint Inc. [69] to fabricate actively matrix-addressable micro-LED display. The electrical connection of the transferred micro-LEDs and micro-ICs is achieved by photolithography and metal deposition processes, and then each micro-LED platelet can be controlled with the corresponding micro-CMOS circuit in an integrated subpixel. Figure 6B and C shows the optical microscope images before and after metal interconnecting, respectively. Although the resolution can be reduced due to the introduction of CMOS circuits in the subpixels, the active-matrix driving mode can effectively improve the brightness of the micro-LED display and reduce the crosstalk between pixels.

In fact, transfer integration is one of the most direct and effective integration methods because most of inorganic micro-devices and their arrays (such as micro-LED, microsensor, and micro-CMOS) can realize homogeneous or heterogeneous integrated devices for multifunctional applications through multiple transfer printing [71], especially for micro-LED display. In addition, transfer integration benefits large-area displays due to the fact that the micro-LEDs array can be expanded by multiple printing processes. Mass transfer technique is considered to be the only way for large-area flat-panel micro-LED displays in the future. Moreover, transfer integration can fulfill full-color displays with wider color gamut and higher efficiency owing to the elimination of color converter. Furthermore, since it is free from rigid substrate, transfer integration has unique advantages in flexible displays.

However, high cost is a major challenge that hinders transfer integration at present. Due to limited yield (<99.9999%) of mass transfer, the repairment and redundancy aggravated large costs [39,41]. In addition, this package-like integration process limits the resolution of micro-LEDs, and transfer integration is hence only suitable for large-scale displays. Moreover, the micro-LEDs with different wavelengths of light require different operating currents, which creates challenges to design driving circuits. Nowadays, a fixed industrial model has been presented as follows: LED manufacturers provide micro-LED chips, silicon fabs provide corresponding CMOS driver panels, and packaging companies assemble them through mass transfer technologies. After a long period of development and patent competition, mass transfer has become an engineering issue with multiple paths, and future breakthroughs in terms of high resolution, high yield, and low cost can be realized with advanced equipment and innovative technology.

## Bonding Integration of Micro-LED

**Fig. 7.**
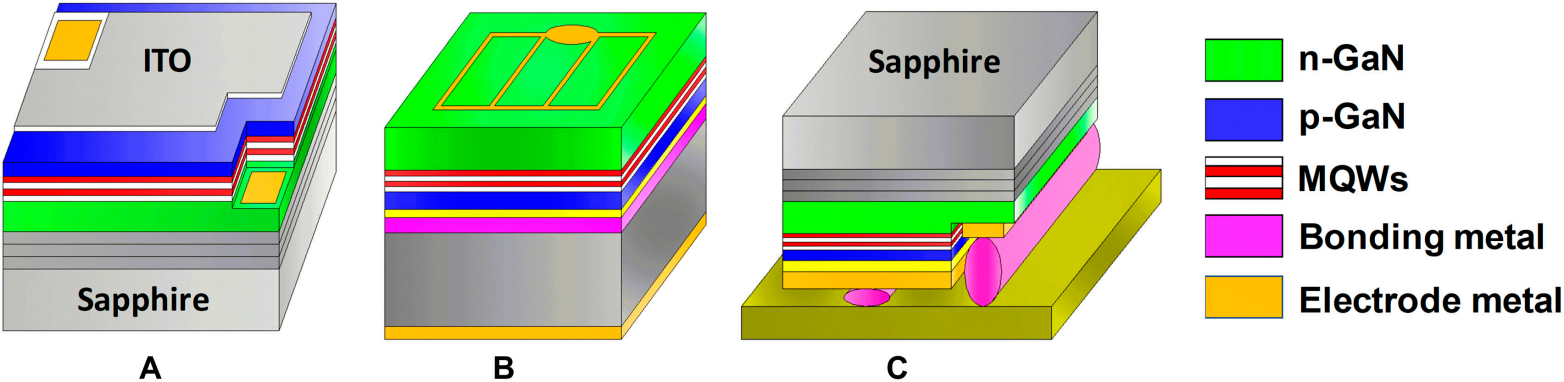
Schematic diagrams of (A) face-up chip, (B) vertical chip, and (C) flip chip.

In 1989, wafer fusion, also called wafer bonding, was proposed for optoelectronic device fabrication and integration [73]. The application of wafer bonding technology in the field of LEDs can be traced back to the bonding of transparent substrates to AlInGaP-based LEDs for higher luminous efficiency [74,75]. Relying on the wafer-bonding process, flip-chip structure and vertical structure LEDs are successively developed from traditional face-up structure LED. The schematics of 3 LED structures are depicted in Fig. 7. Because p-GaN has a lower growth temperature than n-GaN, LED devices generally use p-GaN as the luminous surface, which is the so-called face-up or lateral structure as shown in Fig. 7A. However, the output power and efficiency of the face-up chip are constrained by the uneven lateral current spreading. Vertical structure LED was fabricated by wafer bonding to optimize current spreading due to the vertical current path [76–80], as depicted in Fig. 7B. In addition, the n-face luminescence can improve the light-extraction rate via n-GaN roughing treatments [81]. The Si bonding substrate can optimize the thermal management of the devices. Nevertheless, the electrodes of vertical structure LED is separated on the different side of the wafer, which is not desirable for large-scale integration. Therefore, as a form of package, flip chip was developed to obtain more efficient emission from n-GaN side by wafer bonding [82,83]. Wafer bonding can also be used to fabricate high-performance micro-LED displays, in which the vertical chip and the flip chip are finally presented. Here, we define bonding integration as a type of integration approach of micro-LED, in which the wafer bonding technique was used to integrate discrete devices and materials. On the one hand, the wafer bonding technique was used to assemble multicolor LED to realize full-color displays. On the other hand, the integration of micro-LED and driving circuits was fabricated by wafer bonding.

### Bonding integration for full-color display

**Fig. 8.**
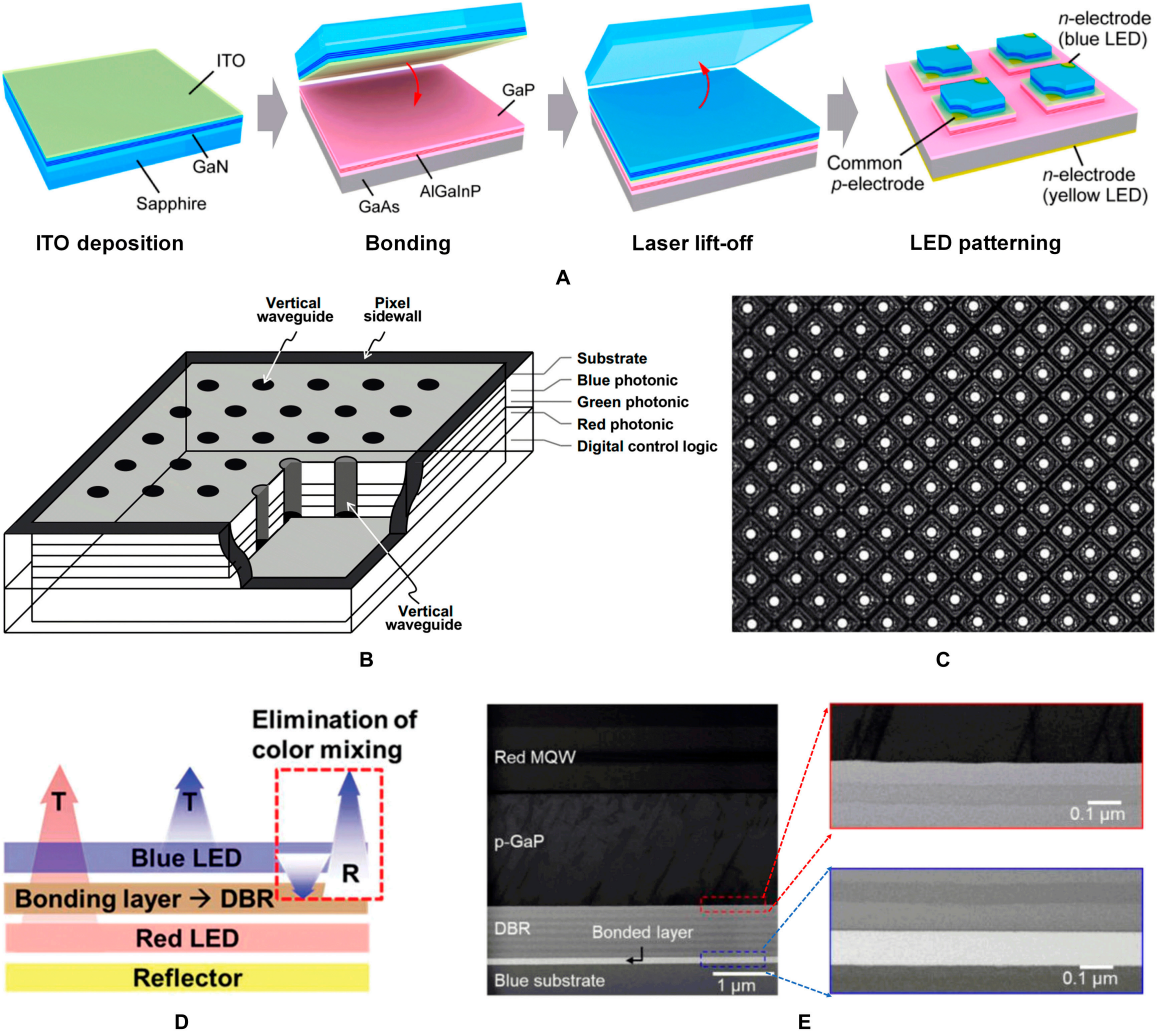
(A) Bonding integration of blue LED epitaxial layer and yellow LED epitaxial layer [84]. (B) Cross-sectional schematic of a full-color micro-LED pixel with stacked RGB active layers and vertical waveguides. (C) SEM image of the full-color micro-LED array for display [85]. (D) Schematic of color mixing principle for polychromatic multiple quantum wells (MQWs) bonded with DBR. (E) Cross-sectional SEM image of full-color vertically stacked micro-LED wafer [86].

In essence, wafer bonding is a process that integrates homogeneous or heterogeneous materials on a single substrate. When red, green, and blue LED epitaxial wafers are bonded on the same substrate, micro-LEDs fabricated from the hybrid LED wafer will have pleochroism. In 2014, Chun et al. [84] tried to fabricate color-tunable LEDs with vertically stacked hybrid wafer via bonding integration as shown in Fig. 8A. Using indium tin oxide layer as an adhesion layer, the GaN-based blue and AlGaInP-based yellow LED epitaxial layers were bonded together. After substrate removal with LLO and device patterning, the integrated LEDs with color-tunable emission were obtained. This work opens the possibility of realizing full-color micro-LED displays with vertically stacked epitaxial materials. However, due to its vertically stacked configuration, the light output of the LED at the bottom is hindered, resulting in a limited light-extraction rate of the device. In addition, the AlGaInP materials with a lower energy bandgap can be excited by high-energy photons emitted from GaN-based LED, which causes severe interference between LEDs in the hybrid chip.

To solve the interference problem and fulfill the intentional color modulation, Ostendo Inc. [85] developed a new approach for full-color micro-LED display. Metal bonding was used to stack the epitaxy wafers with a different primary color wavelength on a patterned CMOS substrate. Afterwards, the micro-LED array was patterned and the pixel pitch was 5 to 10 μm. Then, an array of vertical waveguide structure was designed across through the hybrid pixel for light extraction (Fig. 8B). Using this method, the emitted light of different wavelengths can be coupled in the waveguide to achieve color control, rather than inside the chip. Finally, a full-color display with HD-720 resolution was demonstrated. Figure 8C shows the scanning electron microscopy (SEM) image of the display matrix. Although metal bonding and waveguide coupling can effectively eliminate the interference problem, it comes at the expense of reducing the area of the luminescent active layer. Limited waveguide cross-sectional area also reduces light-extraction efficiency.

An alternative bonding method was proposed by Korea Advanced Institute of Science and Technology [86]. The distributed Bragg reflectors (DBRs) consists of SiO_2_/SiN_*x*_ pairs were used as bonding medium as shown in Fig. 8D. By designing the structure and number of SiO_2_/SiN_*x*_ pairs, the DBR will be capable of selectively reflecting blue light and transmitting red light at bonding interface. Figure 8E shows the cross-sectional SEM image of hybrid wafer. The flat bonding interface proves this method is feasible for manufacturing the ultrahigh-resolution full-color displays. Owing to the designed DBRs, light from each active area in a stacked chip can only be emitted outwards, not inwards. This approach is expected to allow a large light extraction because the light-emitting area is approximately equal to the pixel size. However, fabricating DBRs with high reflection and high transmission will remain a challenge. Mismatched DBR will result in reduced light extraction and color gamut.

**Fig. 9.**
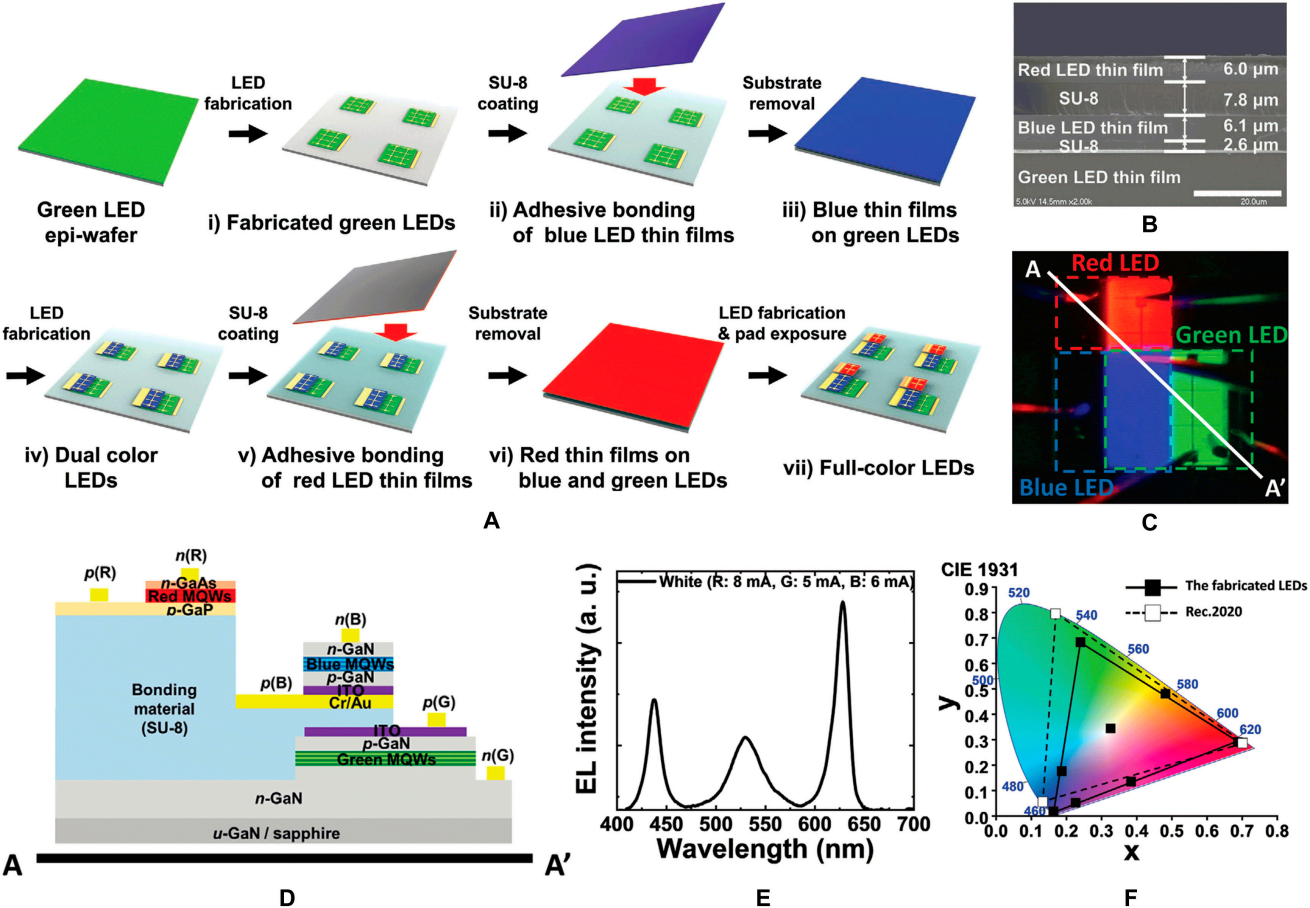
(A) Schematic of the fabrication steps for the full-color inorganic LEDs by the bonding integration. (B) Cross-sectional SEM image and (C) microscope image of the full-color LED. (D) The cross-sectional schematic of the full-color subpixel. (E) EL spectra of the full-color LEDs in white color mode. (F) Commission Internationale de l'Eclairage (CIE) coordinates of the full-color LEDs for various color modes [90].

In addition to vertical stacking, bonding integration also enables lateral configuration of RGB micro-LEDs. Dong-Seon Lee’s group [87–90] developed a method of adhesive bonding by SU-8. The optimized bonding process for full color is shown in Fig. 9A. When the green LED array has been fabricated, a layer of SU-8 was coated on the wafer and then bonded with the blue LED wafer. The growth substrate can be removed by using wet etching. After the blue LED array was competed, the red LED wafer can be integrated by using the same process. Figure 9B shows the cross-sectional SEM image of the full-color wafer, which clarifies the 3 LED films and 2 bonded layers. The luminescence photo and cross-sectional schematic of final full-color subpixel can be seen in Fig. 9C and D, respectively. Figure 9E and F shows the electroluminescence (EL) spectrum and the Commission Internationale de l'Eclairage color coordinates of the full-color LED, respectively. Similar to transfer integration, lateral configuration allows the micro-LED display with wider color gamut and higher light-extraction efficiency. At the same time, the bonding integration enables the micro-LED dies to be directly formed on the wafer by photolithography, potentially achieving ultrasmall pixel size and ultrahigh resolution.

### Integration with drive circuit

Flip-chip bonding has been widely employed for packaging broad-area LEDs, offering LEDs a reverse light exit window for high-efficiency light extraction. For micro-LED, flip-chip bonding facilitated the hybridization process, which consists of fabricating micro-LED array on sapphire or silicon wafer, separately fabricating active-matrix CMOS circuit and hybridizing them by wafer bonding. Hybridizing means the alignment of electrodes and assembly of devices, i.e., each LED pixel should be electrically connected to a dedicated driver subcircuit. For good electrical connection and stable mechanical properties, most of the hybridization process was performed with metal bonding.

**Fig. 10.**
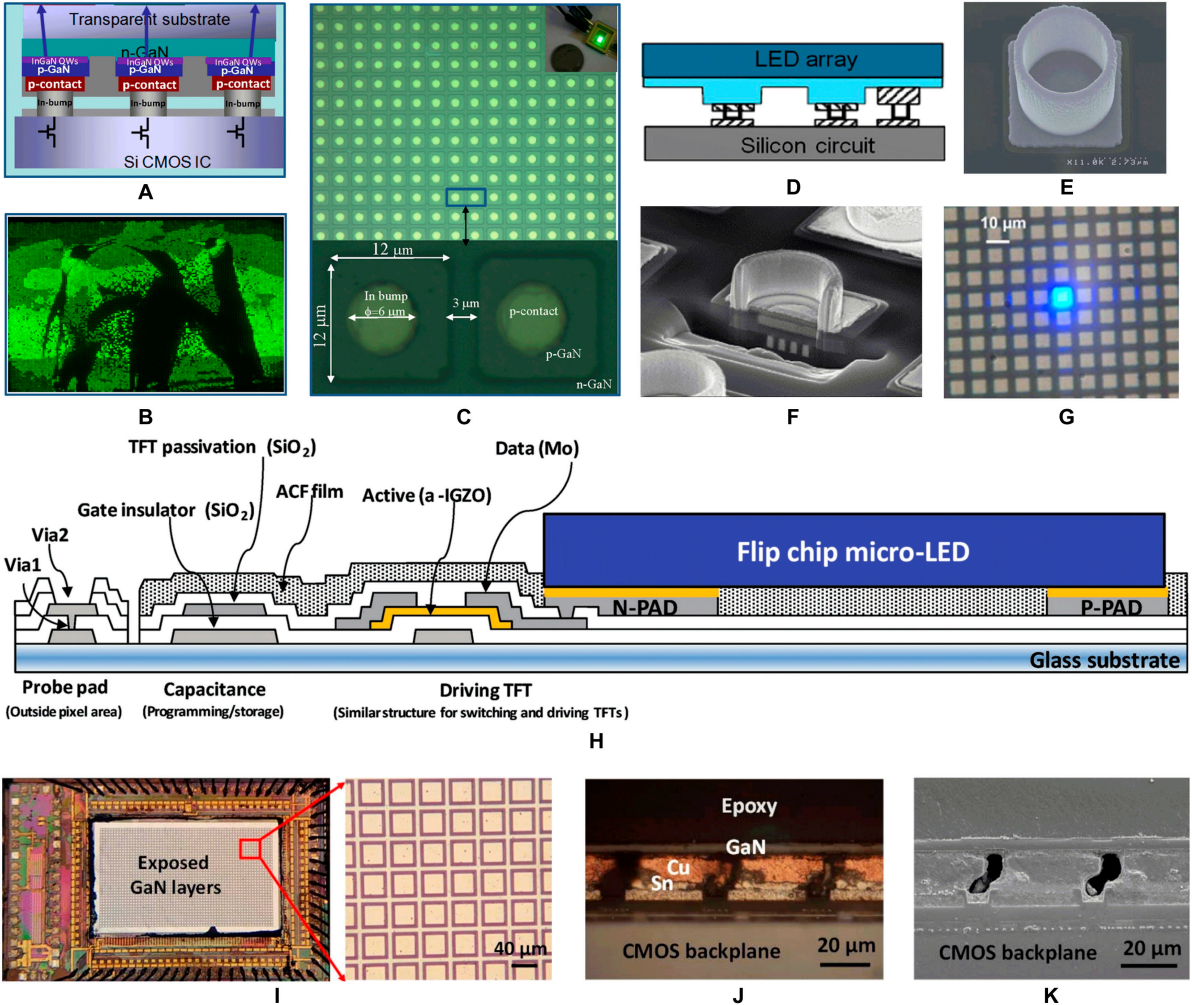
(A) Schematic and (B) physical image of micro-LED display fabricated by flip-chip bonding with In bump. (C) The zoom-in image of a segment of a micro-LED array chip [10]. (D) Schematic of micro-LED bonded by microtube. (E) SEM images of the surface and (F) cross-sectional topography of the microtube [92]. (G) A single micro-LED in a display array emits light driven by CMOS. (H) Anisotropic conductive paste (ACF) film [141]. (I) Integrated chip after Si growth substrate removal and zoomed-in image of the micro-LED array. (J) Optical and (K) SEM cross-sectional images of the Cu/Sn bonding interface [142].

Griffin et al. [91] first demonstrated the micro-pixelated flip-chip LEDs, in which Au bumps were used as bonding metal. Alignment marks were designed on the LED wafer beforehand, and a 16 × 16 array of 72-μm GaN micro-LEDs was hybridized with a Si mount. Based on this research, Day et al. [10] reported a hybrid integration between micro-LED arrays on sapphire and Si CMOS active-matrix ICs (Fig. 10A). The reflow soldering technology were employed for low-temperature bonding with 6-μm-diameter indium bumps. As a result, a high-resolution video graphics array microdisplay (with 12-μm pixel size) was realized (Fig. 10B and C). However, due to the CTE mismatch between LED wafer and CMOS wafer, the reflow soldering process can cause misalignment problems at high soldering temperature.

To solve the problem, CEA-Leti [92–94] developed a microtube technology to achieve flip-chip bonding at room temperature (Fig. 10D), which relieved the thermal mismatch between epitaxial substrate and bonded substrate to the greatest extent. First, microtubes composed of hard metal deposited with Au film was fabricated on the bottom CMOS wafer (Fig. 10E and F). Next, indium bumps were formed on the top LED wafer. As the hard microtubes can insert into the soft indium bumps under a certain bonding pressure, the bottom wafer and the top wafer can be assembled at room temperature without misalignment. In this way, a high-resolution display with sub-10-μm pixel pitch can be achieved, showing great potential in the field of high-end microdisplays (Fig. 10G). Another room-temperature bonding of micro-LED display was reported by Kyung Hee University in South Korea [95]. As shown in Fig. 10H, an amorphous indium-gallium-zinc-oxide TFT array was fabricated on a glass substrate first, and then micro-LED wafers were transferred onto the TFT backplane with flip-chip bonding. The anisotropic conductive paste film with adhesives and Au-plated particles was used as a bonding layer. The driver IC are also bonded and connected to the TFT backplane. A 2-inch active-matrix micro-LED display with high-resolution of 384 × 128 was obtained. Because TFTs can be manufactured in a large area at a low cost, the bonding integration in this work can realize large-area micro-LED displays. However, micrometer-scale Au particles in the adhesives will become uneven when the micro-LED pixel continually decreases. Hence, the uniformity and stability of the micro-LED pixel will be limited by the anisotropic conductive paste film bonding layer.

In addition to misalignment problems, heterogeneous sapphire substrates can also cause severe light crosstalk of flip-chip pixels. However, high-cost LLO process is needed to completely release sapphire substrate. To circumvent these issues, Zhang et al. [16] demonstrated a flip-chip micro-LED display based on GaN-on-silicon epilayers (Fig. 10I), in which a solid Cu/Sn stack bonding layer is used (Fig. 10J and K). Si substrate can be totally removed using a SF_6_-based RIE process. As a result, this bonding scheme enabled the microdisplay to have advantages of non-crosstalk, high stability, and low cost. Moreover, Guo et al. [96] explored micro-LED array on a glass backplane. By flip-chip bonding, the GaN-LED was transferred from a Si substrate to a glass substrate. Besides, SU-8 was used as insulator material and to reduce light crosstalk. This method is favorable for achieving transparent display, but the stress control during bonding is a challenge.

**Fig. 11.**
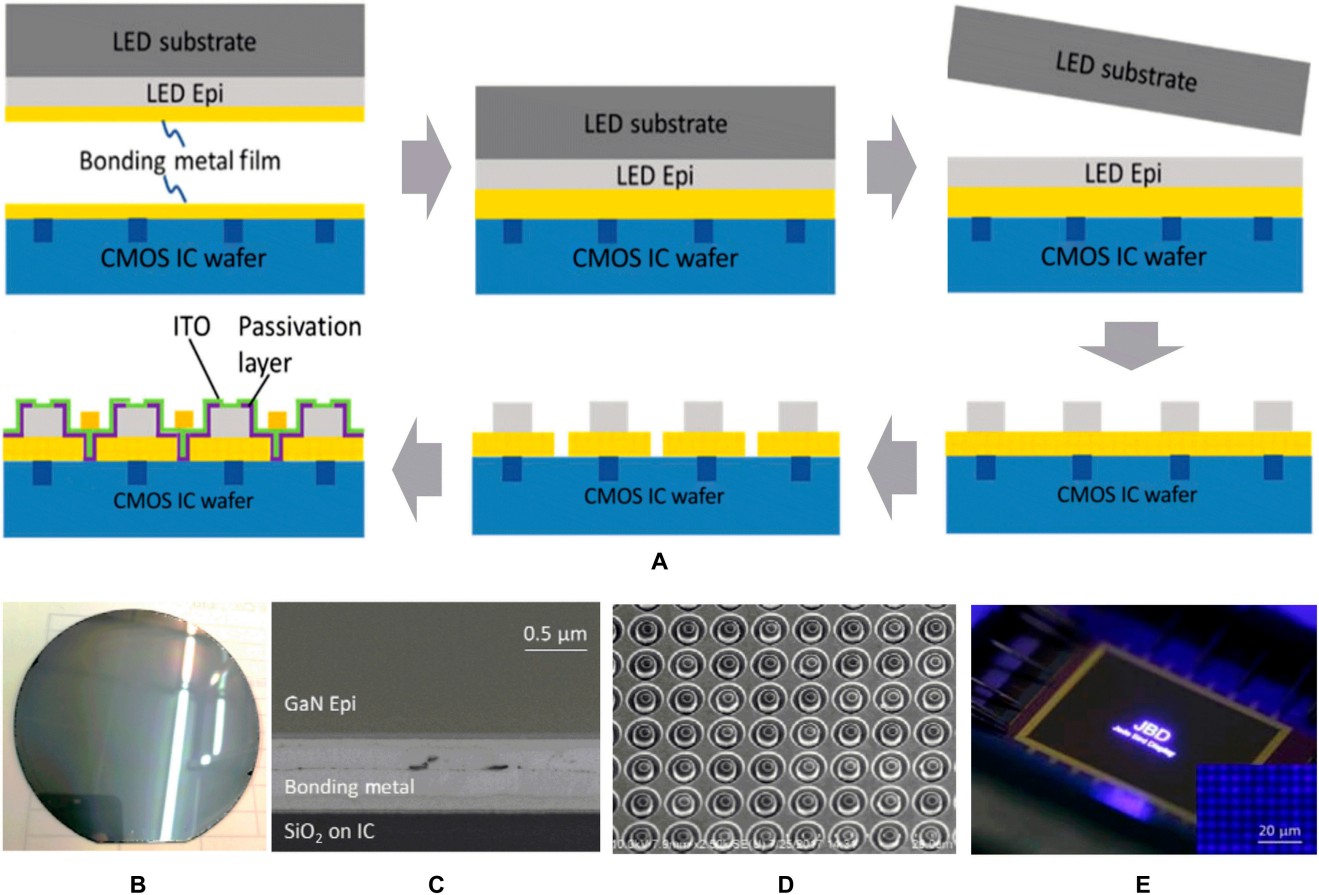
(A) Schematic illustration of the micro-LED microdisplay fabricated by wafer-level bonding process. (B) Picture of a 4-inch GaN epi-on-IC template after the growth substrate is removed. (C) SEM image of the cross-section at the metal bonding interface. (D) SEM picture showing the micro-LED arrays with 5-μm pixel pitch fabricated on the IC backplane. (E) Microdisplays with >5,000-PPI pixel density made in blue colors [97].

Instead of flip-chip bonding a completed array of micro-LEDs, transferring the LED epitaxial layers onto the CMOS wafer can be an alternative integration design. As discussed above, El-Ghoroury et al. [85] has shown a microdisplay by stacking 3-color (RGB) LED epilayers on the CMOS backplane. Due to the CMOS backplane can provide digital control logic and power to the pixels, a truly digital full-color microdisplay device can be obtained. They hence named the device quantum photonic imager. Zhang et al. [97,98] also integrated the 4-inch Si-based IC and III-V epilayers by wafer bonding, and the detailed process steps are described as shown in Fig. 11A. Similar to the preparation method of Si-based vertical structure LEDs [76], the epilayers of LED were transferred to a Si-based CMOS backplane firstly by metal bonding; then, LED chips were fabricated and patterned. It should be noted that the CMOS backplane was specially designed with interconnecting pads through SiO_2_ via holes. During wafer bonding, the epilayers of LED with patterned p-electrodes were aligned and connected to the interconnecting pads. The bonded wafer after removing the growth substrate is shown in Fig. 11B. The cross-sectional SEM image of the bonding interface and the completed micro-LED arrays are shown in Fig. 11C and D, respectively. Through the bonding of epitaxial material and device wafer, an ultrabright monochromatic microdisplay with an ultrahigh resolution (>5,000 PPI) was realized, as shown in Fig. 11E. Due to the stable fabrication process, this scheme of bonding LED epitaxial wafers to CMOS substrates has excellent industrial prospects in high-performance microdisplays. The resolution of such microdisplays depends more on the dry-etching-process accuracy and the control of residual stress in the metal bonding layer

**Fig. 12.**
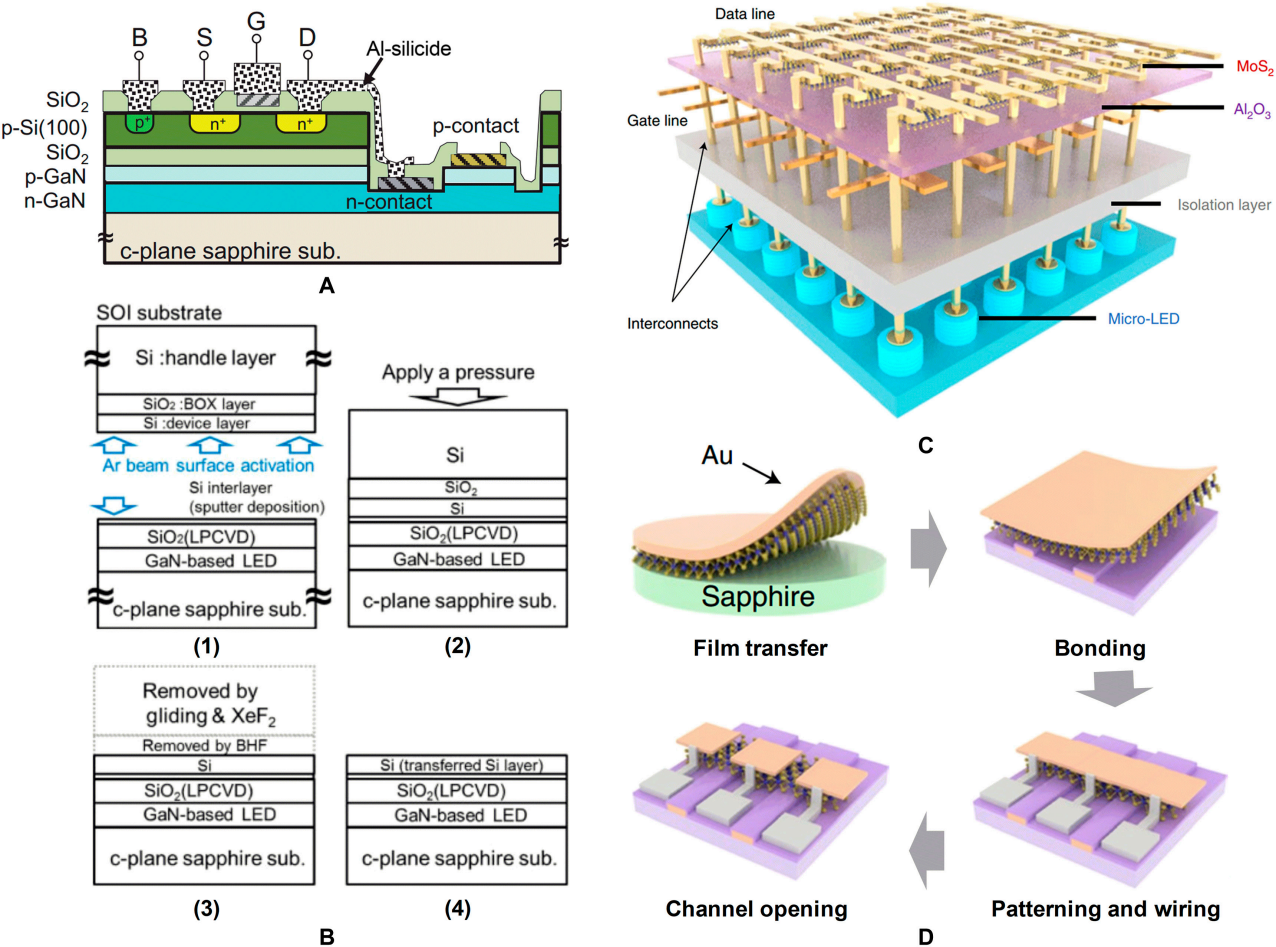
(A) Schematic illustration and (B) process steps of bonding integration of SOI wafer and GaN LED wafer [100]. (C) Schematic illustration and (D) process steps of active-matrix micro-LED display formed by bonding integration MoS_2_ wafer and micro-LED wafer [101].

Integrating the channel material of the transistor with the LED wafer is another feasible process path to realize the integration of micro-LED and its driver circuit Tsuchiyama et al. [99,100] developed a Si/SiO_2_/GaN-LED wafer, as shown in Fig. 12A. A silicon-on-insulator (SOI) substrate was integrated with a GaN-based LED wafer by surface-activated bonding, in which a Si interlayer was embedded as a nano-adhesion layer. The detailed bonding process is shown in Fig. 12B. After removing the Si handle layer from the SOI substrate, the residual bonded Si layer was used to fabricate n-type metal-oxide-semiconductor field-effect transistors (n-MOSFETs), and the micro-LED was exposed by selectively etching. Then, n-MOSFETs were laterally connected with micro-LEDs as a driver circuit for monolithic display system. This bonding approach provides a new idea for realizing monolithic hybrid active microdisplays. Recently, Meng et al. [101] presented a micro-LED display driven by a 2D-material transistor matrix (Fig. 12C). A uniform monolayer of MoS_2_ grown on a sapphire substrate was used as the conduction channel for the drive transistor. A transferred Au film can form intrinsic bonding with MoS_2_ film after mild heating at 90 °C, and then the MoS_2_/Au bilayer can be bonded onto passivated micro-LED array by van der Waals force. The MoS_2_ film was lithographically patterned and dry-etched. The electrode of MoS_2_ transistor can be in-situ formed using the Au film, which can be patterned using water-based KI solution. The detailed process steps are shown in Fig. 12D. Finally, the drive circuit constructed by MoS_2_ transistors was vertically connected through the etching via holes, and a 1,270-PPI active-matrix micro-LED display was demonstrated. This method of directly introducing the channel material to prepare the driving unit simplifies the preparation process of the micro-LED display, but the uniformity of the large-area channel material is the premise of the uniformity of the micro-LED display.

To sum up, whether it is used for full-color display or integrated driving circuits, bonding integration has 2 process paths, i.e., preparing device before and after bonding process. Limited by the alignment accuracy, it is difficult to obtain high-resolution micro-LED displays with the method of preparing devices first and then bonding. If the device is fabricated after bonding, then the pixel size depending on the lithography accuracy may continue to shrink, similar to the scaling down of the CMOS process. Therefore, the bonding-first fabrication techniques benefit the high-resolution and high-throughput micro-LED displays. In addition, the stability of bonding materials and the compatibility with subsequent processes must be guaranteed. Although wafer-bonding integration facilitates the preparation of high-resolution displays, the area of display was inherently limited by the size of epitaxial wafer. Therefore, wafer-bonding integration is suitable for manufacturing small-scale wearable microdisplays.

## Growth Integration of Micro-LED

For Si-based devices, monolithic integration approaches have been applied for constructing system on a chip [102]. As such, instead of wire bonding in package integration, wafer-level electrode interconnections in monolithic integration were desirable for more compact and higher-performance system [103]. In the field of LED, we summarize growth integration into a monolithic integration approach of micro-LED display. The growth integration was defined as an assembly process in which material growth and device preparation are conducted on same wafer. For analogous purposes to Si-based devices, growth integration of micro-LED was beneficial for high frequency and high current density in an ultracompact form factor. In essence, the micro-LED array itself is an example of monolithic integration. Nevertheless, what is discussed in this section is the growth integration of multicolor LEDs for full color and the fully monolithic integration of LEDs and pixel drive transistor circuits.

### Growth integration for full color

Achieving full color with growth integration means that multicolor LEDs are grown on the same substrate. However, the classic red AlGaInP-based LED was challenged to be grown together with a GaN materials platform due to large lattice constant mismatch. Hence, most of growth integrations for full color are currently relied on the assembly of GaN-based LEDs with different colors. On the one hand, as the indium content changes, InGaN/GaN LEDs can theoretically emit light in the entire visible range from blue to red [104]. On the other hand, it was reported that spectrum of c-plane nitride LEDs can be strain-engineered by fabricating nanostructures [105,106]. According to the above principles, growth integration of micro-LED for full color was conducted via 2 types of fabrication techniques, i. e., bottom-up and top-down.

**Fig. 13.**
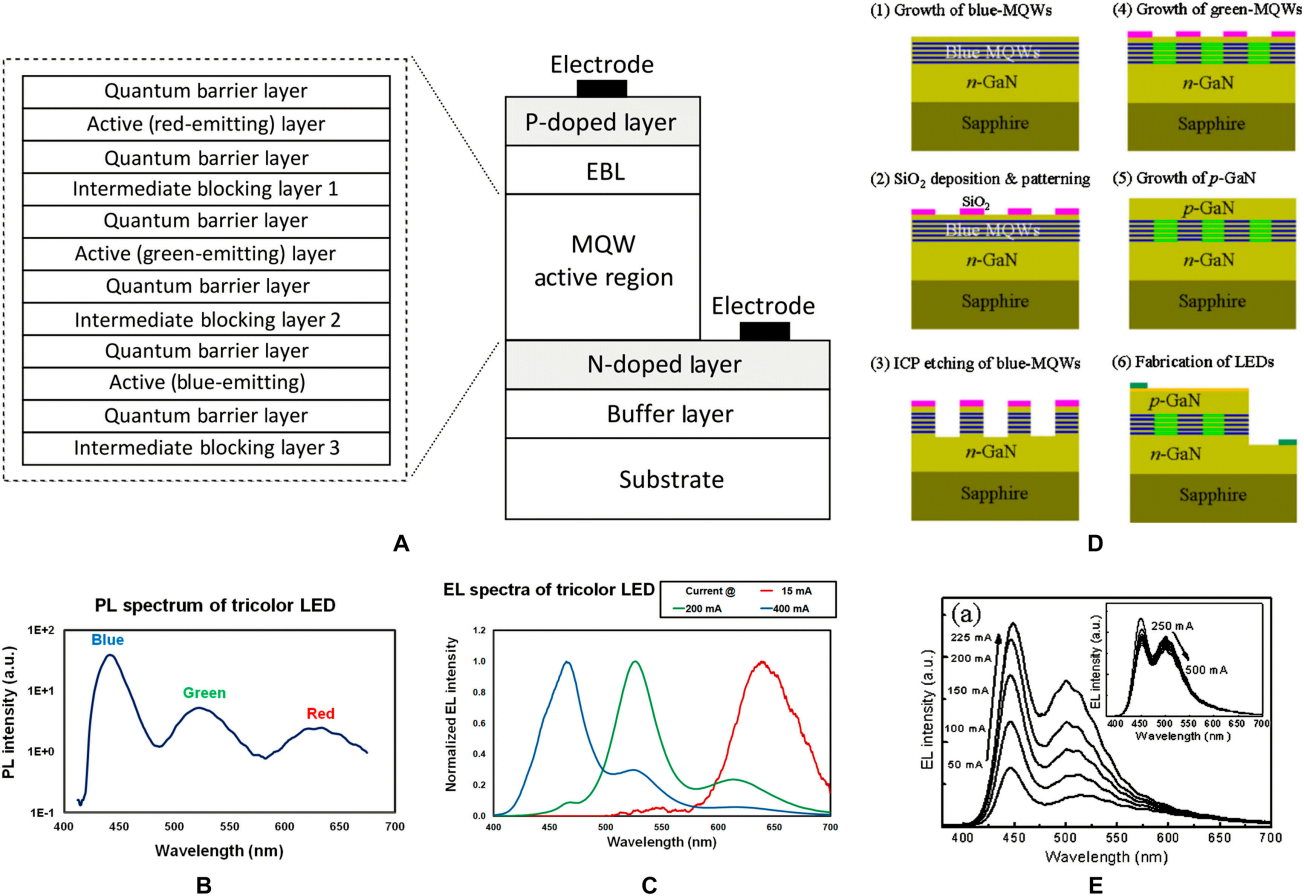
(A) Schematic illustration of tricolor LED wafer with vertically stacked active area. The (B) PL and (C) EL spectrums of the tricolor LED [107]. (D) Fabrication process steps and (E) EL spectrum of LED with laterally distributed polychromatic MQWs [107].

A typical example of bottom-up fabrication techniques is growing polychromatic multiple quantum wells (MQWs), in which monochromatic MQWs were either stacked vertically or distributed laterally. Many studies have reported vertically stacked dichromatic or trichromatic MQWs for broadband GaN LEDs [107,108]. Figure 13A depicts the typical LED structure with vertically stacked polychromatic MQWs for full-color display. The photoluminescence (PL) spectroscopy of the tricolor LED wafer shows the full-color potential (Fig. 13B). However, similar to the bonding integration described above, the stacking configuration creates significant interference between the active regions, which reduces the luminous efficiency and color quality of the device. In addition, the stacked structure will provide insufficient acceptors due to multijunction nature. Because of different distances between those MQWs and common p-electrode, carrier injection and recombination will become uneven. Therefore, the color of the LED depended on the injection current, as shown in Fig. 13C, which will bring great challenges to the design of display driving circuits. To address these issues, Park et al. [109] and Kong et al. [110] demonstrated laterally distributed green and blue MQWs via selective area regrowth as shown in Fig. 13D, which can realize the mixing and modulation of blue and green light (Fig. 13E). On the one hand, due to the lateral configuration or the possibility of independent control by p-GaN isolation etching, quantum wells of different colors can obtain uniform hole supply. On the other hand, the lateral structure can alleviate the interference between the active regions. Even so, it was believed that the abovementioned superimposed growth of polychromatic MQWs complicates material preparation and pixel fabrication. Growing high-quality epitaxial films on uneven wafer surfaces remains a challenge task. Therefore, there are few relevant reports on the preparation of RGB integrated wafers by multistep SAG.

**Fig. 14.**
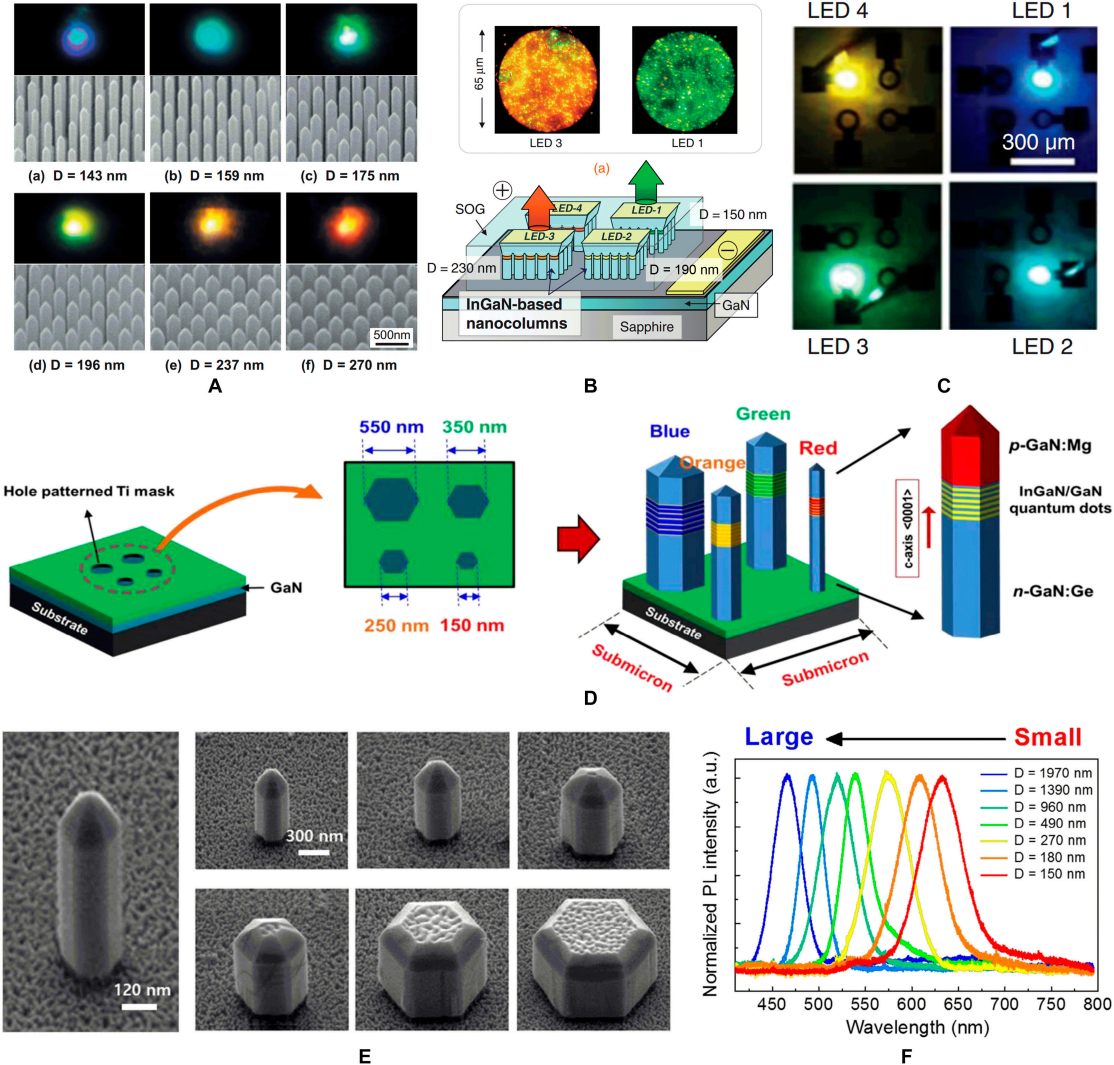
(A) InGaN/GaN nanopillars of different sizes and their emission wavelengths [118]. (B) Monolithic LED nanocolumn array with red and green emissions [119]. (C) Monolithic nanocolumn array multicolor LEDs [120]. (D) Monolithic 4-color pixel integrated by single nanowires. (E) SEM images and (F) PL spectrums of single InGaN/GaN nanowires with various diameters [121].

Instead of superimposed growth, synchronous or 1-step growth was more desirable for growth integration of multicolor LEDs. Different from their flat counterpart, nanowire LEDs with polychromatic emission were reported and can be synchronously grown on the same wafer [110–117]. In 2010, Sekiguchi et al. [118] reported the integrated InGaN/GaN nanowire arrays with blue to red emission, as shown in Fig. 14A. Using electron-beam lithography to pattern Ti film as a mask, nanowire arrays were selectively grown and nanowire diameter was controlled from 137 to 270 nm at an edge pitch of approximately 10 nm. Due to the beam shadow effect of adjacent nanowires and diffusion length difference of Ga and In adatoms on the sidewall, the amount of Ga atoms moving toward the top decreases with increasing nanowire diameter. The decreased incorporation of Ga atoms results in an increased In composition of the InGaN wells. The InGaN quantum well with a higher In component has a narrower bandgap, indicating that large-size nanowire LEDs have longer emission wavelengths. Adopting the above integration technique, Kishino et al. [17,119,120] demonstrated the monolithic integration of green and orange InGaN/GaN nanowire array LEDs in 2013 (Fig. 14B), and monolithic integration of 4-color (red, green, blue, and yellow) InGaN/GaN nanowire array LEDs in 2015 (Fig. 14C). Moreover, in 2016, Ra et al. [121,122] reported a monolithic integration of 4-color (red, orange, green, blue) LEDs with 4 single nanowires instead of nanowire arrays (Fig. 14D). For single-nanowire pixels, beam shadow effect was no longer taken into consideration due to large nanowire pitch. When the nanowire diameter increased, the top of nanowires had larger surface area; then, the lateral diffusion of In atoms was enhanced, which results in a lack of In content and a shorter emission wavelength. As shown in Fig. 14E and F, when the nanowire size decreases from 1,970 to 150 nm, the emission wavelength shifts from the blue to the red band. It is worth noting that the reaction gas flow control and thermal field distribution during selective growth are 2 most important factors to ensure the consistency of device performance, due to epitaxial growth is a process of kinetic and thermodynamic interaction. It was believed that nanowire-based LED exhibits a better luminous efficiency with a lower defect density due to elimination of etching process. However, the nanowire LED pixels faced with 2 challenges. Firstly, In components are difficult to be precisely controlled due to lateral overgrowth of nanowires. Secondly, it is hard to realize electrical interconnection at the pyramidal top of nanowires.

**Fig. 15.**
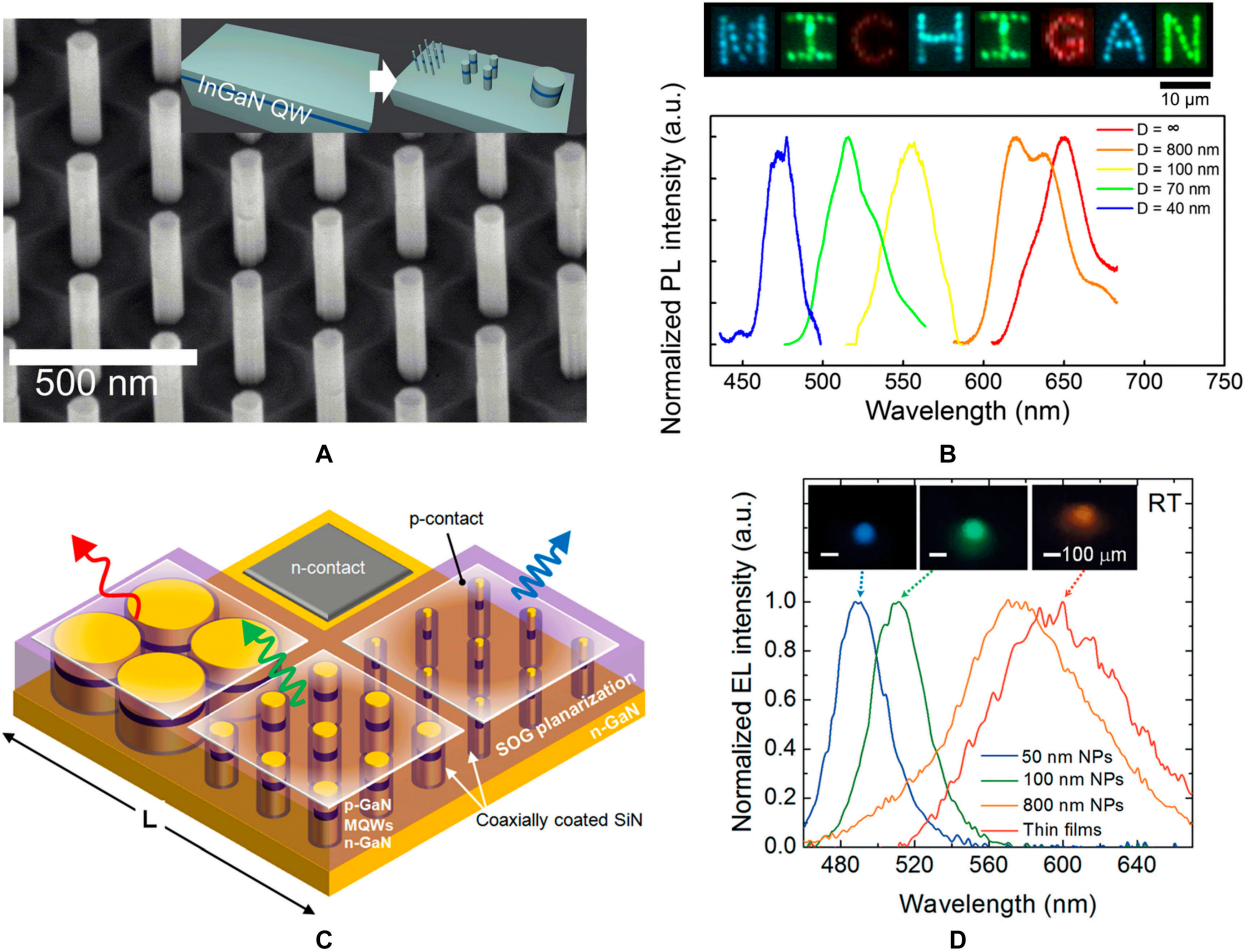
(A) Bird’s-eye-view SEM image of the blue-emitting nanopillar structures consisting of a single InGaN quantum well. The inset shows the schematic of top-down fabrication of nanopillar LED arrays. (B) PL and luminous characteristics of InGaN/GaN nanowires with various diameters [106]. (C) A schematic of top-down fabrication of full-color pixel with various diameters nanopillar LED. (D) EL and luminous characteristics of LED nanowires with various diameters [123].

In order to develop a scalable and manufacturable growth integration for full-color display, top-down fabrication techniques were used to avoid multiple epitaxial steps and selective-area growth. In 2016, Teng et al. [106] reported strain-induced red-green-blue wavelengths tuning in InGaN/GaN MQWs. In their experiment, single red InGaN/GaN MQWs with 32% In components were grown on a c-plane sapphire substrate. Nanopillar LEDs with a diameter from 40 to 800 nm were patterned with electron-beam lithography and RIE, as shown in Fig. 15A. Due to severe quantum-confined Stark effect of the MQWs grown on c-plane GaN, the strain relaxation of etched nanopillars enhances the wavefunction overlap, which leads to their spectral blue-shift and larger emission intensity. Hence, the wavelength tuning will cover all 3 primary colors by engineering the diameter, i.e., strain of the nanopillars. The PL spectrums and luminous pictures of nanowires with various diameters are shown in Fig. 15B. Based on above principles, Chung et al. [123,124] demonstrated the chip-scale integration nanopillar LED multicolor pixels in 2017. As shown in Fig. 15C, each color subpixel consists of an array of nanopillars. It was noted that spin on glass was used for planarizing the nanopillar array, which allowed nanopillars to be compatible with standard planar processes. To better isolate p-contact and n-GaN, the sidewall of nanopillar LEDs was passivated by plasma-enhanced-chemical-vapor-deposited SiN before spin on glass. Figure 15D shows the EL characteristics of the top-down nanopillar LEDs with various diameters. It is reported that the elastic constant of InGaN quantum well depends on the In composition nonlinearly [106]; thus, the strain relaxation and wavelength tuning in this growth integration strongly relies on indium content in the original active region. Theoretically, by adjusting the In doping concentration and optimizing the lithography and etching process, the LED size can be continuously scaled to nano-LED; however, the defect control of the etched sidewall is the key to ensure the electro-optical conversion efficiency of small-size devices.

In summary, growth integration technology includes 2 main process paths to achieve full-color display. Several integration approaches are utilized in these process paths, whose pros and cons have been listed in Table 2. Among them, SAG and SER are considered as 2 competitive approaches to achieve high-performance micro-LEDs and even nano-LEDs. Precise control of growth size, morphology, and doping is the key to the industrialization of the SAG process (bottom-up). Precise control of etch size, uniformity, and improved passivation will benefit the development of SER process (top-down).

**Table 2.** Comparison of bottom-up and top-down full-color techniques for growth integration.

**Fabrication techniques**	**Integration approaches**	**Advantages**	**Disadvantages**
Bottom-up	Polychromatic MQWs	Compact pixel	Nonuniform emission; severe color shift
	Selectively grown nanowires	High luminous efficiency	Inaccurate component control; hard to interconnect
Top-down	Selectively etched nanopillars	Scalable and manufacturable	Etching damage; limited by QCSE

MQW, multiple quantum wells; QCSE, quantum-confined Stark effect

### Growth integration with driving circuit

**Fig. 16.**
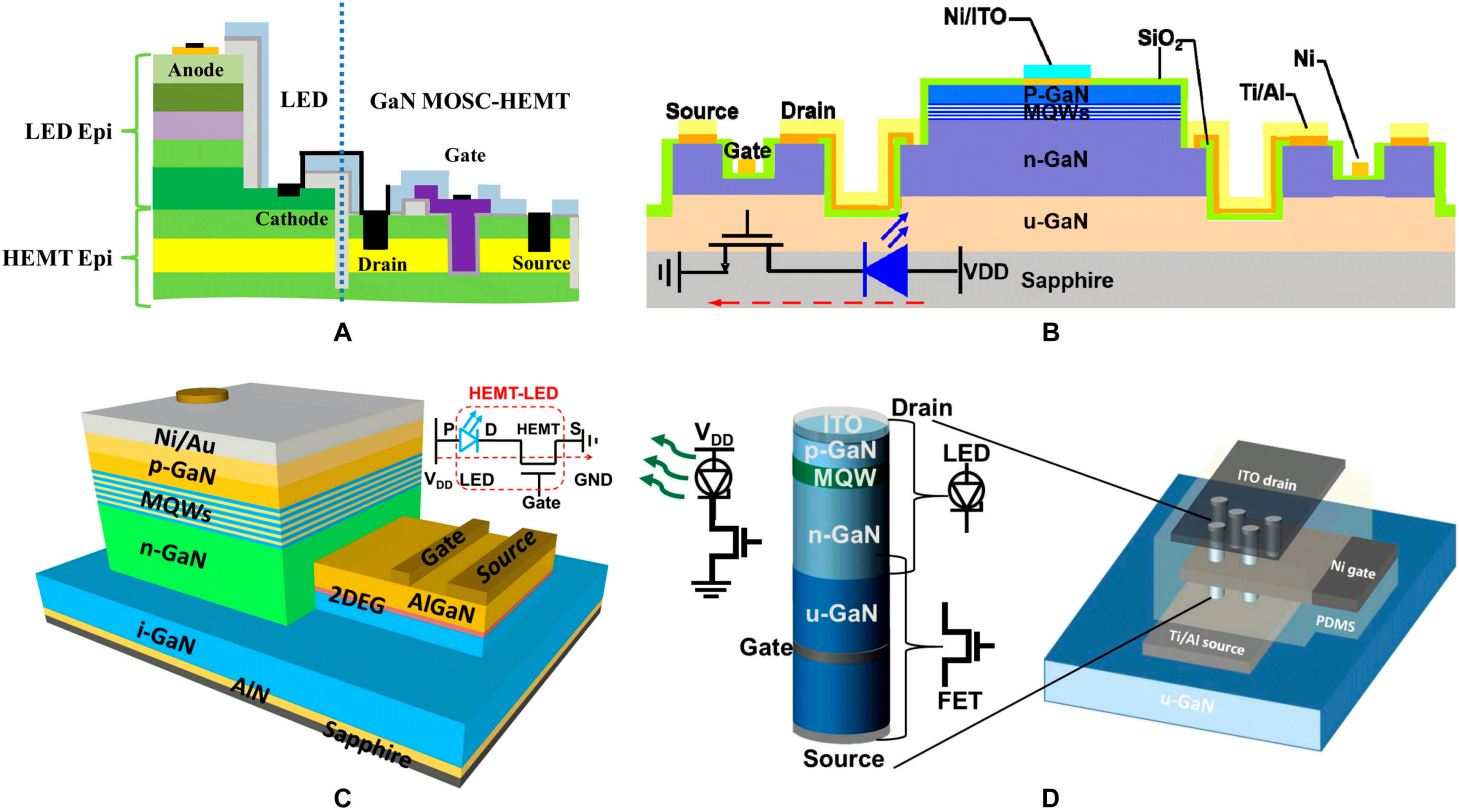
(A) Cross-sectional schematic of the monolithically integrated GaN LED and GaN MOSC-HEMT using the SER approach [126]. (B) Cross-sectional schematic of the monolithically integrated GaN LED and GaN MOSFET [127]. (C) Schematic of the metal-interconnect-free HEMT-LED device using SAG approach [128]. (D) Schematic of the vertical GaN nanowire LEDs with the nanowire FETs [130].

Growth integration is also an effective way to achieve monolithic integration of micro-LEDs and their driving transistors. GaN-based field-effect transistor (FET) was preferred for the driver that can realize in-situ electrical connections to the micro-LED on a common GaN material platform [125]. Li et al. [126] first reported the monolithically integrated LEDs and metal-oxide-semiconductor channel high-electron-mobility transistors (MOSC-HEMTs) in GaN, as shown in Fig. 16A. In their experiment, the LED structure was directly grown on top of the HEMT layers. After selectively etching the epitaxial layer of LED, HEMT was fabricated and serially connected with LED. The light output of LED in this work hence could be modulated by varying the gate voltage of integrated MOSC-HEMT. To simplify the epitaxial structure, Lee et al. [127] reported the monolithic integration of GaN-based LED and lateral GaN MOSFET. Without an additional growth process, MOSFET was directly fabricated using n-GaN layer of LED structure, as shown in Fig. 16B. With SER, the LED structure was trenched to a film of 150 nm for the current channel of the MOSFET. However, it is reported that the process of SER usually causes etching damage to LED structure. Alternatively, Liu et al. [128,129] have demonstrated a superior integrated process with SAG process. Firstly, an as-prepared HEMT structure was selectively etched to 200-nm depth by inductively coupled plasma, exposing the undoped GaN buffer layer and the sidewall GaN channel. Next, the LED structure was selectively grown next to the HEMT structure. Figure 16C shows a finished HEMT-LED device. As n-GaN layer of LED was directly connected with 2-dimensional electron gas (2DEG) generating in HEMT, there will be no need for metal interconnection between LED and HEMT. Thus, this monolithic design facilitated to lower parasitic resistance and smaller device size. Hartensveld et al. [130] demonstrated the growth integration of GaN nanowire LED with GaN-FET. Figure 16D shows the nanowire stacking and the device structure. After a top-down etching, a nanowire LED was formed on the top; meanwhile, a gate-all-around structured GaN-FET can be fabricated using undoped GaN (u-GaN) nanowire as current channel. Instead of lateral integration, the GaN FET and LED in their work was vertically stacked and connected. This vertical stacking structure has multiple advantages, such as no need for regrowth and metal interconnects, free from LED area consumption. However, the GaN gate-all-around-FET in this structure has a large leakage current, which may be detrimental to the control of grayscale and power consumption in display applications

**Fig. 17.**
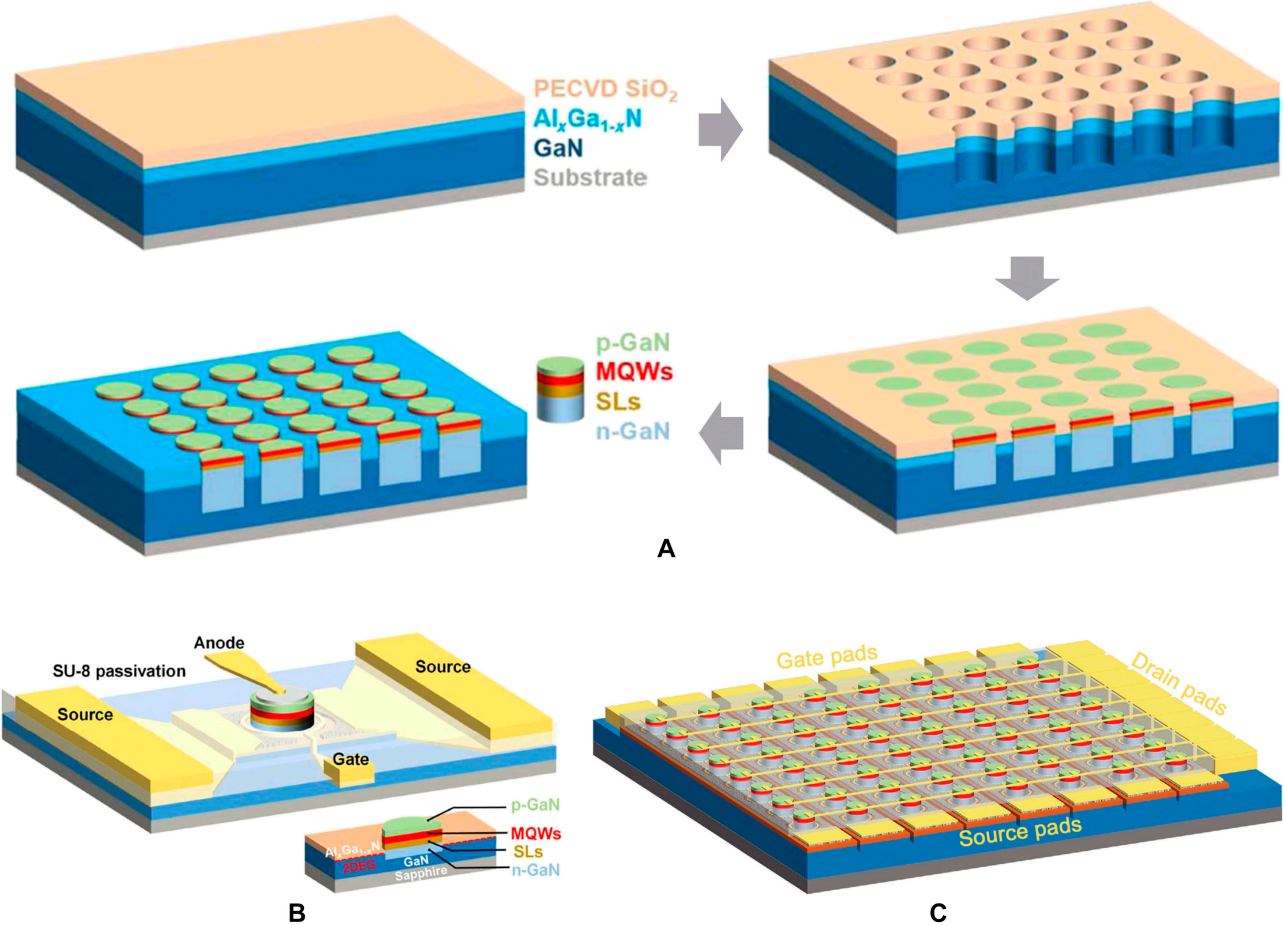
(A) Schematic of the fabrication steps for micro-LED/HEMT hybrid wafer by SAG. Schematic of (B) the fabricated micro-LED/HEMT integrated device and (C) active-matrix display [134].

Although there are many reports on the growth integration of GaN-based LEDs and FETs as mentioned above, these integrated devices have not been applied and fabricated for micro-LED displays for a long time. Recently, monolithically integrated micro-LEDs/HEMTs microdisplay has been reported by The University of Sheffield [131–134]. As shown in Fig. 17A, a SiO_2_ mask was firstly grown on AlGaN/GaN heterojunction wafer by plasma-enhanced chemical-vapor deposition. Then, the selectively epitaxial area was defined by lithography and dry etching. Thus, the 2DEG in the AlGaN/GaN interface was exposed on the etching sidewall. When the micro-LED structure was selectively grown, the n-GaN layer can be electrically connected with the 2DEG. After removing the SiO_2_ mask, a hybrid wafer containing micro-LED and HEMT was obtained [134]. Afterwards, planar processes such as photolithography and etching are used to fabricate integrated devices. The ring-shaped gate electrodes can effectively control the current flow of the HEMT device and drive the corresponding micro-LED in series (Fig. 17B). After matrix interconnection, an 8 × 8 micro-LED microdisplay was fabricated, where the micro-LED size and pitch were 20 and 25 μm, respectively (Fig. 17C). This growth integration scheme eliminates the dependence on heterogeneous integration to a certain extent, making micro-LEDs and their compatible on the same GaN platform. This work paves the way for the realization of all-GaN-based high-performance active-matrix micro-LED displays.

GaN-based transistors as drivers facilitate the growth and integration of micro-LED displays in terms of material compatibility. Nevertheless, 2 process challenges still limit the development of this all-GaN-based micro-LED displays. On the one hand, in consideration of reducing energy consumption and improving circuit safety, the driving circuit tends to use enhancement transistors that work in a normally off mode. However, it is difficult to realize depletion-mode HEMT devices in growth integration. On the other hand, a heat treatment above 800 °C is required for the formation of ohmic contacts during the fabrication of GaN transistors, which will exceed the thermal budget of the LED device and lead to deterioration of device performance.

**Fig. 18.**
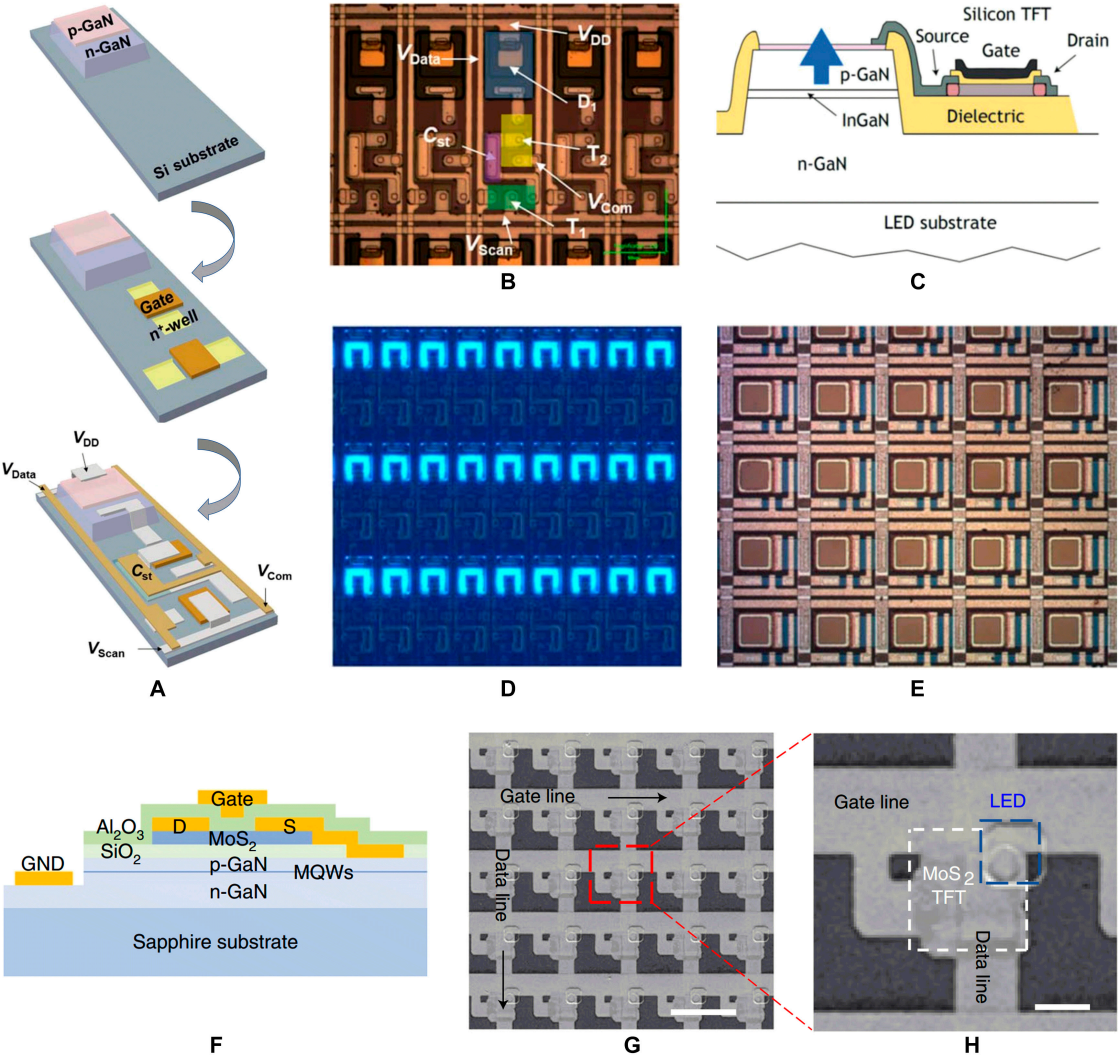
(A) Schematic of the fabrication steps, (B) optical microscope image, and (C) luminous picture of micro-LED display with the drive transistor fabricated on Si substrate [137]. (D) Cross-sectional schematic and (E) top-view microscope image of the monolithic integration of micro-LED and silicon TFT [139]. (F) Cross-sectional schematic of monolithic integration of micro-LED and MoS_2_ TFT. SEM images of (G) the integrated micro-LED array and (H) pixel [143].

For growth-integrating enhancement transistors, Lu et al. [135,136] demonstrated the monolithic integration of GaN-based LED and vertical enhancement GaN MOSFET by selectively regrowing a *p*-GaN and *n*-GaN bilayer on top of a LED structure. Although the *p-n* junction barrier formed by the regrowth can realize the normally off operation of the GaN transistor, it also introduced a larger on-resistance and increased the power consumption of the display unit. Other than pursuing the same material system, Hun et al. [137] prepared Si TFTs on Si substrate directly. After selectively removing GaN LED structure, Si TFTs were fabricated using a standard CMOS process. In their work, a 60 × 60 subpixel monochrome display with 150 PPI was realized. Figure 18A depicts the preparation process flow. Figure 18B and C respectively shows the optical microscope image and luminous picture of final pixel array. This approach can not only exploit the intrinsically enhancement characteristics of Si-based MOSFETs but also facilitate the development of micro-LED display technology by utilizing the mature Si CMOS process.

Thanks to the compatibility design of device structure and fabrication process, Si-based TFT technology, which is widely used to fabricate driver modules for liquid crystal display and organic LED display [138], and Si-based TFTs have also been investigated to monolithically integrate with GaN-based micro-LEDs. Lumiode, Inc. [139] proposed a manufacturable integration scheme using polycrystalline silicon (poly-Si) to form the driving circuits. The micro-LED array was patterned firstly, then a bilayer of silicon dioxide (SiO_2_) and amorphous silicon (a-Si) was deposited to form the active TFT film. In order to increase carrier mobility of TFT channel, pulsed laser annealing technique was used to promote the conversion of a-Si to poly-Si. As the laser beam provided an extremely local, surface-contained treatment, the active layer of micro-LED will not be affected. Next, the integration system can be fabricated with plane CMOS process. Figure 18D and E respectively shows the cross-section and top view of the integrated device. It should be noted that this integration scheme was scalable and suitable for introducing other types of TFTs, such as oxide TFTs [140], 2D material TFTs [141,142], etc. Recently, Hwangbo et al. [143] presented an active-matrix micro-LED display using a MoS_2_-on-GaN epitaxial wafer (Fig. 18F). A 1.4-nm-thick MoS_2_ film was directly synthesized on a 4-inch GaN epitaxial wafer coated with an insulating SiO_2_ buffer layer. The growth temperature of MoS_2_ is as low as 580 °C, which will not cause damage to the active layer of the micro-LED. The SEM image of the finished micro-LED array and a zoom-in image of a micro-LED pixel are shown in Fig. 18G and H, respectively. This approach provides an advanced fabrication pathway for high-performance micro-LED display technology while also challenging the growth of large-scale high-quality 2D materials.

Sharing the same substrate and process flow, growth integration, a fully monolithic integration of micro-LEDs, and driving transistors offers many of advantages. For instance, the power efficiency of driving circuit can be increased by reducing parasitic resistance and capacitance resulted from wire bonding. Besides, using on-chip drivers instead of peripheral components can reduce LED failures and improve the stability of display system, which fully utilizes the long-lifetime superiority of GaN LED chips [39,103]. Furthermore, chip-scale integration can achieve a more compact system with multiple components, fulfilling the advantages of miniaturization and multifunction of ICs. The biggest challenge of growth integration is compatibility of material growth and device process. The management of thermal budget and design device structure play an important role in the integration process. Based on an integrated material platform grown on the same wafer, a variety of device structures could realize the fully monolithic integration of micro-LEDs and driving transistor circuits.

In addition, a series of issues still hindered this integration technology both in terms of full color and driver integration. Firstly, owing to the restraint of wafer size, growth integration techniques cannot be applied to construct large-scale micro-LED displays, whereas, with increasing demand for microdisplays, growth integration was expected to become the preferred technical solution. Secondly, with red-shift spectrum, InGaN/GaN LED has lowering internal quantum efficiency. Therefore, full-color display fabricated by growth techniques had uneven subpixel emissions. Given that, color conversion technique was suggested to realize uniform full-color micro-LED display [144–146]. Thirdly and importantly, lateral growth integration has an inherent contradiction in device design. Generally speaking, the channel of integrated driving transistor should be wide enough to provide sufficient drive current for micro-LEDs. However, the enlarged transistor area will reduce the aperture ratio of subpixels; even the subpixel size can shrink continuously. To resolve this contradiction, TFT with higher carrier mobility and vertical growth integration process should be developed further.

## Summary and Outlook

According to the processing characteristics of micro-LED displays, we have defined 3 integration forms: transfer integration, bonding integration, and growth integration. For each integration form, typical research reports in recent years have been introduced and reviewed. Transfer integration has made great progress in fabricating large-area flat-panel display, while showing excellent potential in flexible wearable displays. With the yield increase and cost reduction of the mass transfer, transfer integration will greatly advance commercialization of micro-LED displays. Bonding integration has unique advantages in achieving high-resolution displays. With precisely aligned wafer bonding, monolithic hybrids of micro-LED array and CMOS driving circuit are expected to realize fully integrated programmable display systems, which benefits portable microdisplays. In addition, growth integration is hoped to achieve fully monolithic systems. With united material platform and process management, growth integration enables a more compact micro-LED display with high efficiency and low energy consumption. Merely, further development of growth integration in micro-LED display needs to address problems of full color and aperture ratio of subpixels. As a matter of fact, a truly practicable display requires the coordination of multiple integration techniques. In the whole system of micro-LED display, complete manufacturing often consists of transferring, bonding, and growing process of materials and devices.

At present, numerous nice prototypes of micro-LED display have been demonstrated by pioneer companies and research institutions. With additional emerging startups, micro-LED display industry is gradually well developed. Although so many studies focused on improving the performance of micro-LED, future integrated displays are supposed to update at many aspects. In terms of materials, large-sized heterogenous epitaxy was desirable for large-scale full-color monolithic displays. In addition to metal-oxide-semiconductor materials, 2D materials that can be mass-prepared will likely be used in the fabrication of driver transistors for future micro-LED displays. Compound quantum dots (QDs) such as ZnS QDs, InP QDs, perovskite QDs, etc. have been integrating with GaN-based micro-LEDs as color-conversion materials in a variety of ways [32,147]. Using blue or UV micro-LED as backlight, the down-conversion of QDs can help to realize full-color display, although this comes at the expense of the micro-LED's luminous efficiency. For structure and process, hybrid integration including multiple integration processes will continue scaling down the micro-LED displays. Differentiated competition will be launched in the field of heterogeneous integration, three-dimensional integration, and flexible integration. Facing future display applications, intelligent display devices is a growing trend. By integrating devices and components with many functions, such as optical waveguides, photodetectors, sensors, actuators, logic and analog circuits, radiofrequency devices and energy harvesters, the smart micro-LED displays will be more widely used in the field of visible light communication, internet of things, and biomedical and micro-nano manufacturing.
